# New Carbon Nanofiber Composite Materials Containing Lanthanides and Transition Metals Based on Electrospun Polyacrylonitrile for High Temperature Polymer Electrolyte Membrane Fuel Cell Cathodes

**DOI:** 10.3390/polym12061340

**Published:** 2020-06-13

**Authors:** Igor I. Ponomarev, Kirill M. Skupov, Olga M. Zhigalina, Alexander V. Naumkin, Alexander D. Modestov, Victoria G. Basu, Alena E. Sufiyanova, Dmitry Y. Razorenov, Ivan I. Ponomarev

**Affiliations:** 1 A. N. Nesmeyanov Institute of Organoelement Compounds of Russian Academy of Sciences, Vavilova St. 28, 119991 Moscow, Russia; kskupov@gmail.com (K.M.S.); naumkin@ineos.ac.ru (A.V.N.); razar@ineos.ac.ru (D.Y.R.); ivan.ponomarev84@gmail.com (I.I.P.); 2 A. V. Shubnikov Institute of Crystallography of Federal Scientific Research Centre “Crystallography and Photonics” of Russian Academy of Sciences, Leninsky Av. 59, 119333 Moscow, Russia; zhigal@crys.ras.ru (O.M.Z.); v.zhigalina@gmail.com (V.G.B.); sufiyanova.alena@gmail.com (A.E.S.); 3 A. N. Frumkin Institute of Physical Chemistry and Electrochemistry of Russian Academy of Sciences, Leninsky Av. 31, bld. 4., 119071 Moscow, Russia; amodestov@mail.ru

**Keywords:** polyacrylonitrile, polymer membrane, polybenzimidazole, electrospinning, membrane-electrode assembly, carbon nanofiber, lanthanides, transition metals, XPS, HT-PEMFC

## Abstract

Electrospinning of polyacrylonitrile/DMF dopes containing salts of nickel, cobalt, zirconium, cerium, gadolinium, and samarium, makes it possible to obtain precursor nanofiber mats which can be subsequently converted into carbon nanofiber (CNF) composites by pyrolysis at 1000–1200 °C. Inorganic additives were found to be uniformly distributed in CNFs. Metal states were investigated by transmission electron microscopy and X-ray photoelectron spectroscopy (XPS). According to XPS in CNF/Zr/Ni/Gd composites pyrolyzed at 1000 °C, nickel exists as Ni^0^ and as Ni^2+^, gadolinium as Gd^3+^, and zirconium as Zr^4+^. If CNF/Zr/Ni/Gd is pyrolyzed at 1200 °C, nickel exists only as Ni^0^. For CNF/Sm/Co composite, samarium is in Sm^3+^ form when cobalt is not found on a surface. For CNF/Zr/Ni/Ce composite, cerium exists both as Ce^4+^ and as Ce^3+^. Composite CNF mats were platinized and tested as cathodes in high-temperature polymer electrolyte membrane fuel cell (HT-PEMFC). Such approach allows to introduce Pt–M and Pt–MO_x_ into CNF, which are more durable compared to carbon black under HT-PEMFC operation. For CNF/Zr/Ni/Gd composite cathode, higher performance in the HT-PEMFC at I >1.2 A cm^-2^ is achieved due to elimination of mass transfer losses in gas-diffusion electrode compared to commercial Celtec^®^P1000.

## 1. Introduction

Development of new electrocatalytic systems which are capable of performing oxygen reduction reaction (ORR) with low overpotentials is of great importance for further progress in polymer electrolyte membrane (PEM) fuel cells (FCs). There are many ways to improve ORR activity of Pt-based catalysts. Among them formation of platinum alloys and Pt–MO_x_ (M = metal) interactions are the most promising. Increased ORR activity of PtNi alloys has been researched in many works [1]. The Pt_3_Ni (111) surface is the most active for ORR in low-temperature (LT-PEMFC) and high-temperature polymer electrolyte membrane fuel cell (HT-PEMFC) membrane electrode assemblies (MEAs) [2]. Several reasons for activity increase of Pt–M (M = Ni, Co) alloys were suggested. The most important of them is the shortening of Pt–Pt interatomic spacings [3], the downshift of the d-band center [4], when alloying modifies Pt electronic structure [5], and better chemisorption of OH groups onto transition metal sites [6]. Alloying inhibits chemisorption of oxygen intermediates, as it correlates to Pt–Pt bond shortening [3]. When the metal binds the oxygen species strongly, the ORR is inhibited by O and OH species present on the surface. When the metal binds oxygen weakly, the ORR is inhibited by slower electron and proton transfer to the adsorbed oxygen. Hence, the ORR electrocatalyst design should be optimized [1,6].

It was found that size and Pt/Ni ratio of Pt–Ni octahedra have a crucial effect on their activity. For example, different compositions of alloys were studied from Pt_3_Ni to PtNi and PtNi_3_, where Pt_3_Ni was found to be the most optimized [1,7]. The size effect is not very clear; however, in general, particles of 6–9 nm are reviewed to be the most optimal [1]. Another review suggests approximately 3–5 nm as the state-of-the-art, considering highly active species up to 20 nm in size [6]. The ways of PtNi octahedra growth appeared to influence multi-metallic composite formation for both shape stabilization and core-shell structures and to lower Ni leaching [1]. Alloys with lanthanides Pt–Ln (where Ln = La, Ce, Sm, Gd and some others) occurred to be useful as ORR catalyst in Pt_5_Ln form [8,9]. The effect of the concrete lanthanide is difficult to predict. From one side, a lattice parameter and d_Pt-Pt_ decrease as atomic mass of Ln increases which enhances electrocatalytic activity. At the same time, their stability decreases with higher strain and lower d_Pt-Pt_ (higher Ln atomic mass) [10]. Platinum–lanthanide oxide catalytic systems Pt–M_2_O_3_/C (M = Ln) are of special interest for electrocatalysis and PEMFC applications [11]. For example, higher ORR activity was found for Pt–CeO_x_/C compared to Pt/C [12]. Also, positive effect of cerium was found for Pt–CeO_2_/C compared to Pt/C in low-temperature PEMFC on cathode side [13]. Enhancement of the ORR kinetics on Pt-Gd_2_O_3_/C compared to Pt/C was observed without any Pt electronic modification in Pt-Gd_2_O_3_/C. Higher ORR kinetics makes this cathode a promising candidate for application in PEMFC [14].

An approach to combine lanthanides and transition metals leads to perovskite-type oxides LnMO_3_, where M stands for first-row transition metals (FRTMs) showing catalytic and electronic properties of special interest [15]. Early-transition metals, such as zirconium, are also applicable in the electrocatalytic systems, for example, PtZrO_2_/C catalyst was found to be higher in stability compared to Pt/C in PEMFC applications [16].

The HT-PEMFC is an important type of hydrogen–air fuel cell operating at 150–200 °C which uses a polybenzimidazole/phosphoric acid-type membrane and is able to consume hydrogen, contaminated by CO [17,18,19,20,21,22,23,24]. Current approaches for electrode production are based on Pt/C electrodes, where platinum is deposited on carbon black mixed with Teflon^®^ and surfactant to make catalytic ink which is sprayed onto carbon paper or carbon cloth. A lot of studies report that carbon black is not stable under acidic high temperature conditions at high potentials. So that, replacement of carbon black by more durable materials, for example, single and multi-walled nanotubes, nanocages, carbon nanofibers (CNF) especially prepared by pyrolysis of electrospun polymer nanofiber mats is really a challenge [25,26,27]. Most of Pt–M and Pt–MOx electrocatalyst systems with high performance are developed for different carbon black supports. But their incorporation into CNF mats is a separate task and requires additional technics.

Earlier we have shown [28,29,30,31,32,33,34,35,36,37], that platinized highly macroporous CNF self-supporting mats, which were obtained by the method of electrospinning (ES) from polyacrylonitrile (PAN) polymer solution [38,39] with further stabilization in air, pyrolysis under vacuum [40], and platination in hexachloroplatinic acid solution, can be successfully used in HT-PEMFC as gas diffusion electrodes (GDEs), both on cathode and anode sides. Thus, polybenzimidazole membrane PBI-OPht was developed in our group for this purpose [41,42]. Initial addition of metal (Zr, Ni) salts into electrospinning polymer dopes allows to reach a uniform distribution of metals in the whole nanofiber, either in metal or oxide form [30]. Particularly, we have shown that GDEs based on self-supporting carbon nanofiber materials, such as Pt/CNF/ZrO_x_/Ni composites, possess high catalytic properties and electrochemical stability when tested in HT-PEMFC. It was shown that Zr is present in composites in a form of oxide and nickel is reduced to Ni^0^ according to XPS. Lanthanide introducing into composite materials may further improve catalytic properties of GDEs created by us for FC applications.

Our approach to produce durable highly porous electrospun CNF based HT-PEMFC electrodes has been recently recognized as effective finding for fuel cells technology [43].

The aim of this study was the screening of new possible ingredient combination and concentration in composites under investigation. All steps starting with preparation of polymer dopes containing metal salts were followed by an open surface electrospinning method producing non-woven mats and their pyrolysis under suitable operational conditions to attain uniform distribution of inorganic additives. Further nanocrystalline Pt deposition on the composite makes it possible to use the produced electrocatalysts for ORR in HT-PEMFC and allows to expand such an approach to improve fuel cell characteristics.

In this study we show the possibility for Pt–M and Pt–MOx electrocatalytic systems to be incorporated into CNF mats instead of carbon black by using the electrospinning method. Also, we show the advantages of these CNF materials when used in HT-PEMFC at high current densities due to the fact of the elimination of mass transport losses. A combination of the different abovementioned approaches will be used, showing the increased electrochemical activity and stability of the catalyst by inserting Gd and Ce into CNFs. Both Gd and Ce are supposed to be in the oxide form: Pt/CNF/ZrO_x_/CeO_x_/Ni and Pt/CNF/ZrO_x_/GdO_x_/Ni, respectively. Also, we present an attempt to insert different lanthanide and FRTM into CNFs, with cobalt and samarium chosen for Pt/CNF/SmO_x_/Co material formation.

## 2. Materials and Methods

### 2.1. Electrospinning and CNF-Based Composite Obtaining

#### 2.1.1. General Data

Electrospinning for all solutions was performed at 26–30 °C, with a relative humidity 8–12%, a distance between electrodes of 180–190 mm, a linear carrier velocity of 3 mm s^−1^, and a carriage velocity 200–250 mm s^−1^ on a Elmarco Nanospider^TM^ NS LAB setup (Elmarco, Liberec, Czech Republic). Electrospinning was processed at 70–80 kV and 0.06 μA. As a result, composites PAN/metal salts nanofiber precursor mats were obtained. Elemental composition was determined with C,H,N-analyzer with thermal desorption column Vario micro cube (Elementar, Langenselbold, Germany). The metals were determined by X-ray fluorescence using X-ray fluorescence spectrometer VRA-30 (Carl Zeiss, Oberkochen, Germany). Electrical conductivity was measured by a four-terminal sensing method with a digital setup RLC E7-8 (Kalibr, Minsk, Belarus).

#### 2.1.2. CNF/Zr/Ni/Gd Composites

Carbon black (0.2 g) Vulcan^®^ XC72 (Cabot, Boston, MA, USA) or simply Vulcan was sonicated in 30 g of N,N-dimethylformamide (DMF) at 50 °C for 4 h to form a stable suspension. Then 2.5 g of PAN (M_w_ 150 kDa) was added to the above suspension under mechanical stirring and allowed to dissolve the polymer. A three-component solution of 0.1 g nickel (II) chloride hexahydrate (NiCl_2_·6H_2_O), 0.16 g of gadolinium (III) chloride hexahydrate (GdCl_3_·6H_2_O), and 0.015 g zirconium(IV) chloride (ZrCl_4_) in 3 g of DMF was mixed with the PAN/Vulcan suspension under intense stirring and sonicated in ultrasonic bath for 3 h at 50 °C. Then, this composite solution was electrospun. In order to obtain CNF/Zr/Ni/Gd composite (sample 1), the polymer composite mat was oxidized at 350 °C in air and pyrolyzed at 1000 °C under vacuum. Further treatment of the sample 1 at 1200 °C under vacuum results in another CNF/Zr/Ni/Gd composite (sample 2).

#### 2.1.3. CNF/Sm/Co Composite

Solution of 2.5 g of PAN (M_w_ 150 kDa) in 30 g of DMF was mixed with the solution of 0.326 g of cobalt (II) chloride hexahydrate (CoCl_2_·6H_2_O) and 0.1 g of samarium (III) chloride hexahydrate (SmCl_3_·6H_2_O) in 2 g of DMF and sonicated in ultrasonic bath for 3 h at 50 °C. Then, this composite solution was electrospun. To obtain CNF/Sm/Co composite (sample 3), the polymer composite mat was oxidized at 330 °C and pyrolyzed at 1200 °C.

#### 2.1.4. CNF/Zr/Ni/Ce Composite

Carbon black (0.2 g) Vulcan^®^ XC72 was sonicated in 30 g of DMF at 50 °C for 4 h to form stable suspension. Then, 2.5 g of PAN (M_w_ 150 kDa) was added to the above suspension under the mechanical stirring and allowed to dissolve the polymer. A three-component solution of 0.4 g nickel(II) acetate tetrahydrate (Ni(CH_3_COO)_2_·4H_2_O); 0.05 g of cerium (III) chloride heptahydrate (CeCl_3_·7H_2_O) and 0.03 g zirconium(IV) chloride (ZrCl_4_) in 5 g of DMF was mixed with the PAN/Vulcan suspension under intense stirring and sonicated in ultrasonic bath for 3 h at 50 °C. Then, this composite solution was electrospun. To obtain CNF/Zr/Ni/Ce composite (sample 4), the polymer composite mat was oxidized at 350 °C and pyrolyzed at 1000 °C.

Electrical conductivities and elemental composition for the samples are given in Table 1.

### 2.2. CNF Platination

Platinum deposition on pyrolyzed nanofiber mats (1, 2, 3, and 4) with area of 6.76 cm^2^ was performed separately for each mat in 10 mL of ultrapure water (Millipore) which contained 0.5 g of HCOOH and calculated amount of H_2_[PtCl_6_]·6H_2_O to obtain a material (Pt/1, Pt/2, Pt/3, and Pt/4) with 1.0–1.2 mg cm^−2^ of deposited Pt. After Pt deposition the materials were left for 3 days in the solution. Finally, they were dried at 100 °C under vacuum for 2 h.

### 2.3. Fuel Cell Testing

Platinated CNF samples were hydrophobized by immersing them into Teflon^®^ AF solution in hexafluorobenzene with concentration of 1 mg mL^−1^ for 1 min. The obtained hydrophobized materials were annealed at 300 °C under vacuum, and the resulting material was ready for use as cathode in membrane-electrode assembly (MEA) HT-PEMFC. As a reference sample, the standard carbon black (Vulcan^®^XC-72)-based cathode Celtec^®^-P Series 1000 MEA [44] was employed. Performance of the MEAs was studied using standard test cells furnished with two graphite plates (Arbin Instruments, College Station, TX, USA) at 180 °C. Flow fields were engraved in the plates. Standard commercial anodes Celtec^®^-P Series 1000 MEA with Pt concentration of 1 mg cm^−2^ deposited on carbon black (Vulcan^®^ XC-72) [44] were used in MEA HT-PEMFC. Polybenzimidazole PBI–OPht membrane [41,42], crosslinked by Zr(acac)_2_, doped by *o*-phosphoric acid (400%, up to 25 of H_3_PO_4_ molecules per polymer chain unit), which was earlier developed in our group (see Supplementary Materials for details in polybenzimidazole PBI–OPht synthesis and membrane obtaining [41,45], Figure S1 from the Supplementary Materials) was used in MEA. The MEA consisted of an anode, a membrane, and an entirely platinated pyrolyzed CNF mat cathode. The assembled MEA was clamped in the test cell between two graphite plates. The MEA polarization curves were recorded at 180 °C and ambient pressure. Flow rates of gases and cell temperatures were controlled by fuel cell test station G-40 (Hydrogenics, Mississauga, ON, Canada). Membrane-electrode assembly of 5 cm^2^ working area was placed in the test cell. A Potentiostat Elins P-150X (Electrochemical Instruments, Chernogolovka, Russia) was used as the electronic load. During the testing, the anode and cathode of the cell were supplied with dry hydrogen (100 mlpm) and dry air (600 mlpm), respectively. Cell voltage was scanned at the rate of 5 mV s^−1^ in the voltage range from 0.95 to 0.1 V. It took at least five consecutive voltage cycles for stabilization of the performance curves. In fuel cell tests all gases were used at atmospheric pressure. Sample Pt/1 after its work in fuel cell is mentioned as Pt/1’.

### 2.4. Electron Microscopy

The solution with CNF was applied onto standard copper grids with a holed amorphous carbon film and subsequently dried under normal conditions. The structure of nanocomposites was investigated by the methods of scanning electron microscopy (SEM) using an FEI Scios microscope (FEI, Hillsboro, OR, USA), transmission electron microscopy (TEM), high-resolution transmission electron microscopy (HRTEM), scanning transmission electron microscopy with a high-angle annular dark-field detector (HAADF STEM), electron diffraction, energy-dispersive X-ray analysis (EDX), and elemental mapping using a FEI Titan 80–300 microscope and a FEI Tecnai Osiris (FEI, Hillsboro, OR, USA) with accelerating voltage of 200 kV equipped with a special SuperX EDS system including four silicon detectors for rapid obtaining of chemical distribution maps. Electron microscope images were analyzed using Digital Micrograph (GMS 3, Gatan, Pleasanton, CA, USA), Esprit (Esprit 2, Bruker, Billerica, MA, USA), TIA (TIA 16, Siemens AG, Munich, Germany) and JEMS software (P. Stadelmann JEMS—EMS Java version 2004 EPFL, Lausanne, Switzerland).

### 2.5. X-Ray Photoelectron Spectroscopy

The X-ray photoelectron spectra were recorded using an Omicron spectrometer (Omicron NanoTechnology, Taunusstein, Germany) and a Sphera II hemispherical electron energy analyzer with Mg and Al Kα radiations in the fixed analyzer transmission mode. Survey and high-resolution spectra of appropriate core levels were recorded at pass energies of 160 and 40 eV and with step sizes of 1 and 0.1 eV, respectively. The energy scale of the spectrometer was calibrated to provide the following values for reference samples (i.e., metal surfaces freshly cleaned by ion bombardment): Au 4f_7/2_ 83.96 eV, Cu 2p_3/2_ 932.62 eV, Ag 3d_5/2_ 368.21 eV. Sample area of 5 mm × 700 mm contributed to the spectra. The residual pressure inside the analysis chamber was lower than 5.0 × 10^−9^ mbar. The photoelectron spectra were approximated by Gauss function or the sum of Gauss functions, and the background caused by secondary electrons and photoelectrons that lost energy, was approximated by Shirley-type background line. Quantification was performed using atomic sensitivity factors included in the software of the spectrometer. The samples were mounted on a sample holder with a two-sided adhesive tape. The spectra were collected at room temperature. Sample charging was corrected by referencing to sp^2^-state deconvoluted in the C 1s spectrum (284.44 eV) [46]. In the absence of sp^2^-state, C–C/C–H state was used with assignment energy of 284.8 eV.

### 2.6. Cyclic Voltammetry

Electrochemically active surface area (ECSA) of platinum in the synthesized electrocatalysts was evaluated by electrochemical hydrogen adsorption/desorption measurements [47,48]. Hydrogen atoms on Pt surface were produced by electrochemical reduction of H^+^ from aqueous 0.5 M H_2_SO_4_ electrolyte. The number of surface atoms at polycrystalline Pt was evaluated from the charge associated with oxidation of adsorbed hydrogen in the potential range 0 to 0.4 V versus standard hydrogen electrode (SHE), using the stoichiometry of one adsorbed H atom per single surface Pt atom:

H_3_O^+^ + Pt_s_ + e^−^ = Pt_s_H_ads_ + H_2_O

Measurements were done at room temperature in a three-electrode cell with separated compartments. Pt wire and Ag/AgCl-saturated KCl (0.2 V versus SHE) were used as a counter and reference electrodes, respectively. The working electrode was a polished graphite disk of 1.6 cm^2^ diameter in a PTFE holder. A thin layer of the catalyst under investigation coated the surface of the disk electrode by the repeated placing aliquots of the catalyst ink with intermediate drying at 60 °C. Catalyst inks were prepared by ultrasonically dispersing 2–3 mg of a catalyst in 0.4 mL of aqueous solution containing 0.2 mL of isopropanol and 0.01 mL of 5 wt.% Nafion solution. 100 µL of the ink was dispersed onto the surface of the disk electrode, fifty cycles of voltammetry at 50 mV s^−1^ were applied and the last cycle was examined. The ECSA was evaluated by integration of the hydrogen adsorption/desorption areas of the cyclic voltammograms (CV) assuming 0.21 mC(cm_Pt_)^−2^. The dashed areas which were used for integration are shown in Figure S2. Capacitance of the double layer was taken into account. On the voltammograms, current readings are normalized per unit of Pt loading at the graphite disk electrode.

## 3. Results and Discussion

### 3.1. Electrospun CNF Composite

The method of electrospinning provides a lot of opportunities to obtain nanofiber composite materials with homogenous distribution of particles of different nature including metals, for example, first row transition metals and lanthanides. The most promising modern ES method from free surface (Nanospider^®^) was used in this work and made possible to obtain several square meters of nanofiber composite materials through a wide range of thicknesses (5–200 μm) and fiber diameters (50–300 nm). Application of the method of electrospinning to the dopes containing PAN and metal salts resulted in a homogeneous distribution of the inorganic additives. After carbonization of the PAN matrix during pyrolysis, metal salts transformed into reduced metal or/and oxidized MO_x_ form. Finally, after Pt deposition, it results in the distribution of platinum electrocatalyst and helped to optimize Pt surface area and, consequently, resulted in ORR optimization. Electrospun polyacrylonitrile nanofiber with distributed metal salts acted as (nano) reactor where metal and M^n+^ may participate in many reactions of PAN precursor forming metal/C composites. As a result, Pt–M/C and Pt–MOx/C catalytic systems could be formed, avoiding additional metal or metal oxide deposition on the surface of carbon material which prevents metal leaching from the system. Such an approach allows to introduce Pt–M and Pt–MOx (previously known to be deposited on carbon black) into CNF mats. In this case, metal and metal oxide particles are embedded into carbon nanofiber material matrix making them more stable to leaching. Introduced nickel also acts as carbon crystallinity (graphitization) center which increases during pyrolysis. The possible mechanism of the effect could be related to nickel–carbon interactions which trigger lessening of a carbon lattice distortion by graphitization [49].

### 3.2. CNF/Zr/Ni/Gd

**Figure 1:**
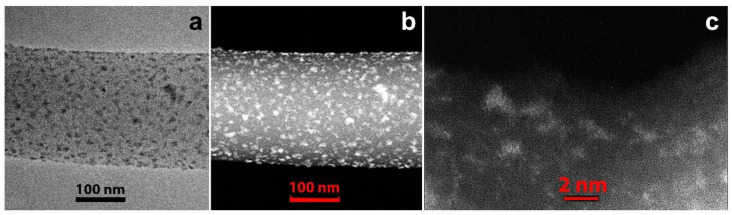
Pyrolyzed CNF/Zr/Ni/Gd composite 1: (**a**) bright field transmission electron microscopy (BF TEM) image; (**b**) scanning transmission electron microscopy with a high-angle annular dark-field detector (STEM HAADF) image; (**c**) STEM HAADF image of small particles.

**Figure 2:**
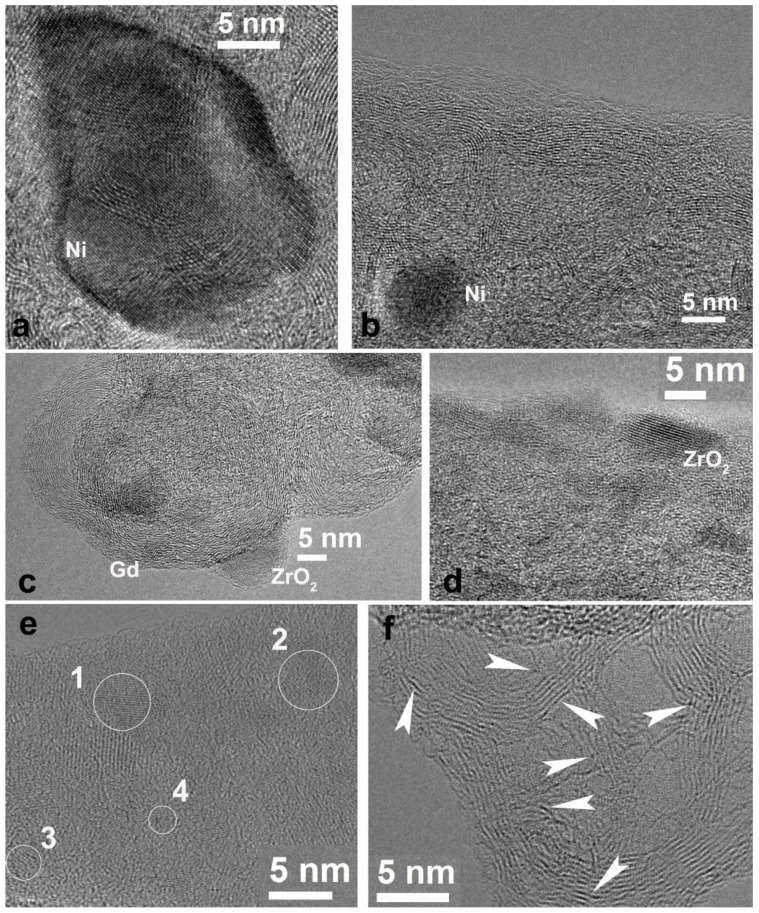
HRTEM images of pyrolyzed CNF/Zr/Ni/Gd composite 1: (**a**) Ni particle of a large size; (**b**) Ni particle of a small size; (**c**) metal particles, grown on a surface of the onion-like carbon black particles; (**d**) ZrO_2_ particle on the surface of a CNF; (**e**) metal nanoparticles in a thin carbon layer (1–3: Gd/Gd_2_O_3_ and 4: ZrO_2_); (**f**) atomic clusters, atom chains, and isolated atoms of metal (shown by white arrows).

To obtain CNF/Zr/Ni/Gd composite material 1, zirconium (IV) chloride, nickel (II) acetate, and gadolinium (III) chloride were added to PAN/DMF solution containing Vulcan^®^XC-72 (5 wt.%) suspension for subsequent electrospinning. After the abovementioned process, the resulting nanofiber mat was oxidized at 350 °C in air and pyrolyzed at 1000 °C under vacuum. The images of a separate nanofiber with metal particles are shown in Figure 1 and Figure 2.

The detailed investigation (Figure 1a) showed that the nanofiber surface was uniformly covered with particles of different size. The largest particles reached dozens of nanometers (Figure 1b), while the smallest ones reached 1–2 nm (Figure 1c). The HRTEM image (Figure 2a) and corresponding Fourier diffractogram analysis indicated that the large particles were those of Ni^0^ (their interplanar distances d_hkl_ according to HRTEM images are 2.04 and 1.77 Å). All particles were of random shape. Conjugated atomic planes of the particle and a nanofiber close to the particle are clearly visible in Figure 2a which shows quasi-epitaxial growth of the particles due to a proximity between some interatomic distances of Ni and carbon nanofiber.

**Figure 3:**
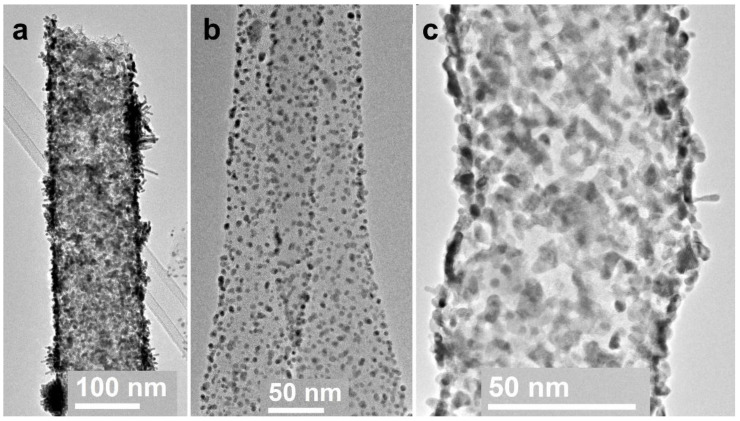
TEM images of Pt morphology for platinated pyrolyzed composite 1: (**a**) needle-like; (**b**) spherical; (**c**) intermediate (random).

Many of the Ni particles were of a few nanometers in size and mostly round in shape (Figure 2b). In the same figure, a crystal structure of pyrolyzed nanofibers is clearly seen. The parallel graphene layer sets form loops and pores of nanometer size. Structures of that kind, described in some of our previous studies [29], appear after pyrolysis at high temperature and illustrate the degree of CNF crystallinity. In Figure 2c, one can observe a typical carbon black onion-like particle with nanoparticles of Gd (or Gd_2_O_3_) and ZrO_2_. The image illustrates metal and/or metal oxide particles on carbon black inclusions integrated into the CNF. They can be formed due to the interactions between metal salts and carbon black in the spinning dope. Nanoparticles of Gd and Zr oxides of 2–3 nm are found on the surface of the nanofibers as well (Figure 2d). The structure of these particles depends on various defects in the crystal lattice (twins, monatomic steps). A lot of particles were also well visualized on thin parts; “films” of carbon black, being detached from the fibers and located near CNF (Figure 2e). According to HRTEM interplane distance measurements, the particles denoted as 1–3 in Figure 2e are Gd (or Gd_2_O_3_) (P63/mmc, *d_hkl_* =1.80–1.83 nm); 4 is a particle of ZrO_2_ (P2_1_/c) with atomic interplane distances d_hkl_ = 1.81 and 1.66 nm. However, the metals in the composites did not exist only in the form of nanoparticles with well-formed crystalline structure. Atomic clusters of 1 nm in size, atom chains located between parallel graphene layers in nanofibers, and even isolated atoms were seen and shown by arrows in Figure 2f. Therefore, structural investigations of the composite samples before platination show uniform metal and metal oxide nanoparticle distribution on the CNF surface. Platinum was deposited from aqueous solution of H_2_[PtCl_6_] in the presence of formic acid as a reducing agent. TEM images of platinated nanofibers are shown in Figure 3.

**Figure 4:**
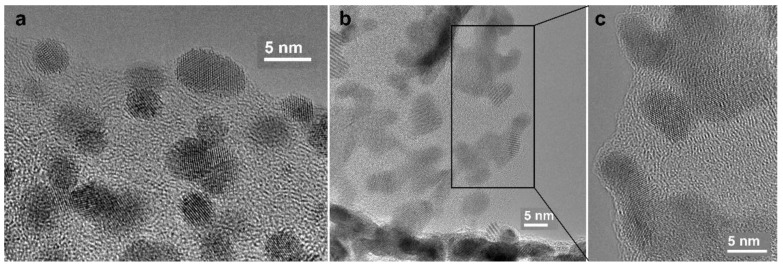
HRTEM images of Pt nanoparticles for platinated pyrolyzed composite 1: (**a**) spherical morphology; (**b**) intermediate (random) shape; (**c**) enlarged image of selected area in (**b**).

Platinum nanoparticles, as can be seen in Figure 3, were not scattered uniformly on the surface of nanofibers. Different platinum morphologies were observed, namely, needle-like (Figure 3a), spherical (Figure 3b), and an intermediate form of Pt (Figure 3c). Spherical and intermediate forms prevailed in the sample. As it can be seen for composite 1 (Figure 3), the length of the platinum needles reached values up to 50 nm (Figure 3a). The HRTEM images of the spherical and intermediate forms of Pt are shown in Figure 4.

**Figure 5:**
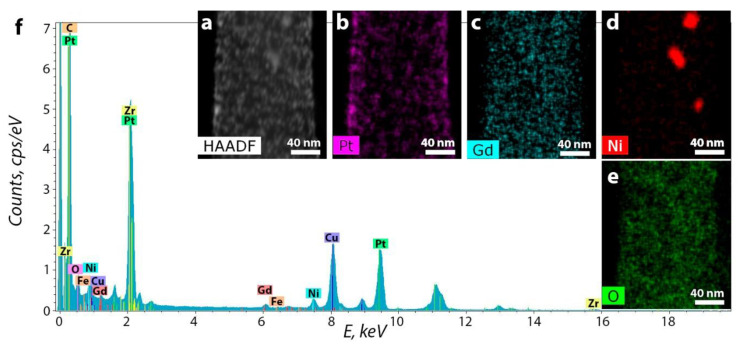
(**a**) HAADF STEM image; energy-dispersive X-ray spectroscopy (EDX) distribution maps for (**b**) platinum, (**c**) gadolinium, (**d**) nickel, (**e**) oxygen; (**f**) EDX spectrum for platinated pyrolyzed composite 1.

The particle size of the spherical Pt was a few nanometers, representing intergrowths of nanocrystals with many defects such as vacancies, twins, and monoatomic steps on the surface (Figure 4a). Particles of intermediate form represent monocrystal intergrowths (Figure 4b). Pt nanoparticles are located in the CNF surface layer. Element distribution maps of zirconium, nickel, and gadolinium in the nanofiber composite material and platinum deposited on it are shown in Figure 5.

The presence of copper peaks in all EDX spectra was due to the use of copper grids with a carbon substrate. On the one hand, these results are in good agreement with the data on metal nanoparticles obtained by the TEM and HREM methods. On the other hand, the data on the distribution of elements make it possible to evaluate the location of platinum and other metals relative to each other. All metals and oxygen are uniformly distributed on the CNF surface. It can be noted that no explicit correlation in the arrangement of nickel, gadolinium, and platinum was observed. Besides, Ni^0^ also forms large particles of dozen nanometers in size which have no oxide layers as it was mentioned before. Homogeneous distribution is needed for higher efficiency and better performance of the material in fuel cells as it helps to enlarge electrocatalyst surface area and correlates to ORR optimization and better reproducibility of the material [1,6].

**Figure 6:**
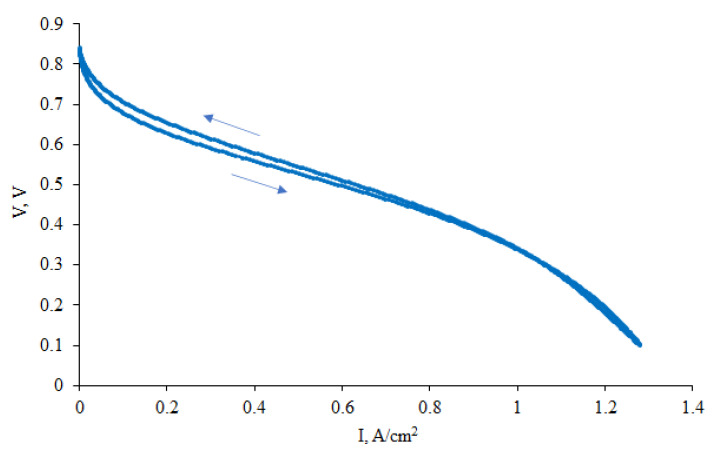
Polarization curve for membrane-electrode assembly (MEA) with platinated CNF/Zr/Ni/Gd composite cathode Pt/1.

Pt’s electrochemically active surface area in the synthesized catalysts was evaluated by the electrochemical method of hydrogen adsorption/desorption on Pt as described in Section 2.6. Some of the respective cyclic voltammetry curves are shown in the Supplementary Materials, Figure S2. The dashed area on the curves was used for integration of the charge ascribed to oxidation of adsorbed hydrogen. Pt’s electrochemically active surface area was in agreement with the high performance of the fuel cell (Figure 6).

**Figure 7:**
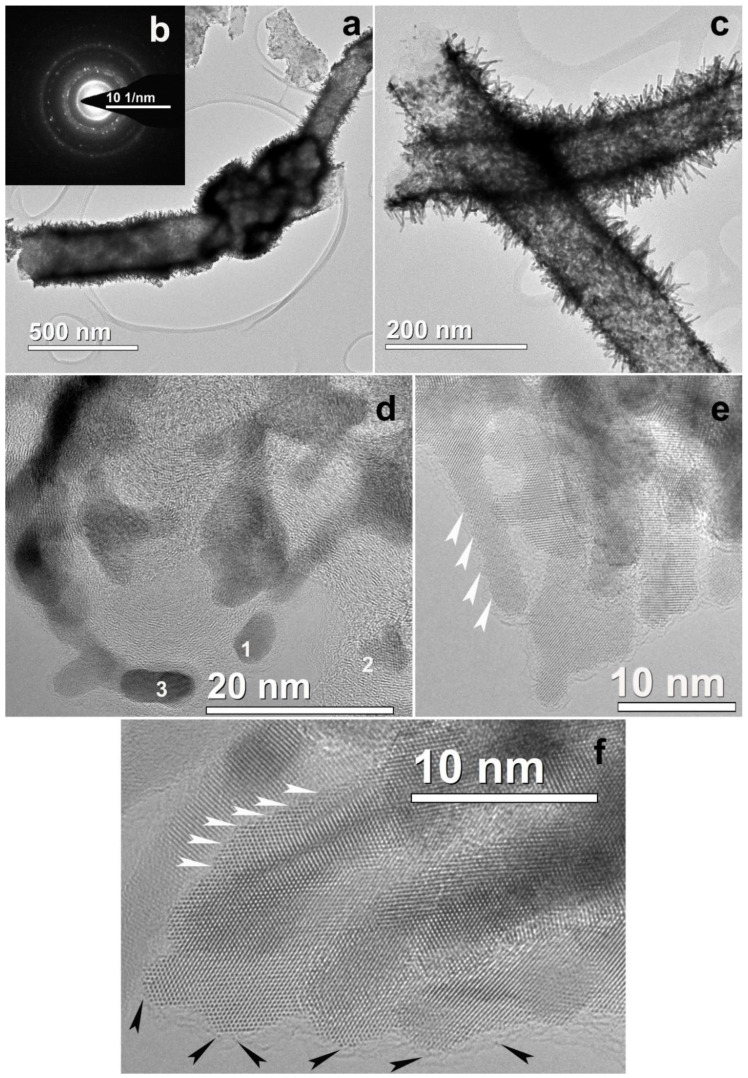
Structure of platinated composite Pt/2: (**a**) CNF with carbon black and (**b**) corresponding selected area electron diffraction (SAED) pattern; (**c**) needle-like long Pt on CNF; (**d**) carbon black influence on Pt particle morphology (1,2—Pt particles; 3—Ni particles); (**e**) HRTEM image of needle-like Pt with monoatomic steps; (**f**) fine structure of Pt crystals of intermediate (random) shape. Ledge-steps are shown with white arrows, isolate atoms are shown with black arrows.

For the first time it was determined that for carbon composite material even before and after Pt nanoparticles deposition when pyrolysis temperature increases up to 1200 °C during composite synthesis, morphology and distribution of all elements change unexpectedly. TEM images of platinated composite nanofibers Pt/2 are shown in Figure 7.

Figure 7 shows that a carbonization temperature increase of up to 1200 °C brings about growth of Pt nanocrystals in a needle-wire form up to 50 nm in length. For this sample such morphology of Pt prevails. At the same time, needle-type platinum is not formed on some of the nanofibers. Spherical particles of 2–15 nm were observed as well. In addition, nanofibers with different platinum concentration are seen. “Puffiness” of the nanofibers can be explained by the presence of carbon black integrated in carbon composite material (Figure 7a). Pt crystals of different morphology are shown in HRTEM images (Figure 7d–f). Needle-like crystals possess many steps (shown by white arrows). Needle-like platinum is monocrystalline. The orientation of such platinum nanocrystals is mostly the same; they grow almost perpendicular to the CNF axis. HRTEM images reveal crystals with (111) planes perpendicular to their growth direction (longer axis of a nanocrystal). The analysis of Fourier diffraction patterns shows that Pt needles have face-centered cubic (fcc) lattice-like bulk platinum crystals [29]. Isolated atoms (shown by black arrows) are located close to Pt crystal surface with ledge-steps. The fact is that nucleated rounded particles of random Pt form with smooth contours surround integrated onion-like carbon black on CNF surface. According to HRTEM images (Figure 7d), Pt nucleation on CNF happens as follows. Firstly, separate metal atoms assemble into chains and elongate along bundle planes (such bundles are shown in Figure 2). Secondly, these primary chains determine Pt crystal growth and its basis morphology [50,51,52]. Rounded forms of Pt particles which repeat the onion-like shape of carbon black are clearly seen in Figure 7d. The same growth mechanism likely happens for other metals and their oxides. After additional annealing (pyrolysis), the CNF’s crystallinity degree increased which promoted more intensive Pt particle growth in the form of needles. A growth mechanism of nanocrystals with fcc lattice along the most packed <111> direction can be explained by the minimum energy principle. For crystals of the intermediate shape, isolated Pt atoms around the edges are attached to them. These atoms on the edge of crystals are shown with black arrows in Figure 7f. Steps and terraces are shown with white arrows in Figure 7e,f. It was previously supposed that the catalytic activity in ORR was determined by the atomic structure of the surface of the nanoparticles, namely, its step nature. The authors of Reference [53] showed that in oxidation reactions, the activity of particles of platinum or its alloys with a size of about 2 nm in size is determined by crystal lattice sites located on the steps of nanoparticles, while activity in ORR is determined by the sites of the crystal lattice located on the surface of the terraces (100) and (111).

**Figure 8:**
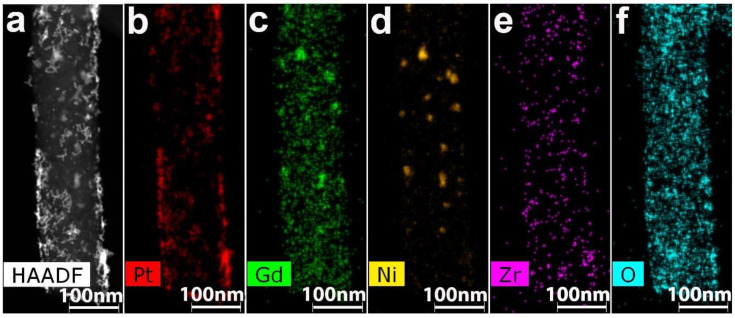
(**a**) HAADF STEM image; EDX distribution maps for (**b**) Pt, (**c**) Gd, (**d**) Ni, (**e**) Zr and (**f**) O for the Pt/2 composite.

The EDX distribution maps for Pt/2 are shown in Figure 8.

As it is seen in Figure 8, large-size particles of Gd and Ni coincide on the maps. At the same time, a coincidence of Gd and Zr nanoparticles is practically absent. According to EDX spectra, on some parts of nanofibers, Ni and Gd are present almost in the same concentrations. On the other parts of material Ni may prevail Gd or vice versa. Zr and O are distributed uniformly and may coincide. According to distribution maps, Gd and Ni also coincide in large particles. At the same time, Ni is located in the core, and Gd is located in the shell.

**Figure 9:**
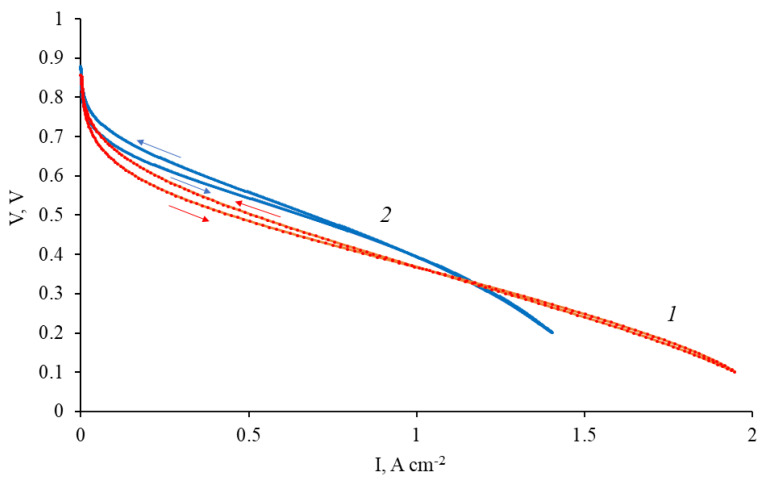
Polarization curves for the Pt/2 cathode (1) and commercial Celtec^®^P1000 cathode (2).

The polarization curve for the composite material Pt/2 used as cathode in the membrane–electrode assembly with PBI–OPht membrane at 180 °C is shown in Figure 9.

As it is seen from Figure 9, the performance of Pt/2 cathode is somewhat below than the one for commercial electrode at current densities <1.2 A cm^−2^ where the main sources of losses are those of activation losses and ohmic ones. However, at current densities >1.2 A cm^−2^ (where the main source of such performance is mass transfer losses) the performance of Pt/2 cathode is surprisingly higher than that of commercial one. It can be related to higher macroporosity of such type CNF materials (up to 90% of free volume, according to our previous studies [29]) compared to the performance of the ink sprayed Pt/carbon black electrode. High macroporosity prevents “flooding” of the cathode which usually happens at high current densities due to massive release of water during electrochemical ORR in the fuel cell. Such advantage is found for the Gd-containing CNF based electrode, pyrolyzed at 1200 °C.

The performance of MEA with composite Gd-containing nanofiber cathode (Pt/2) was better than with non-composite, pure carbon cathode (pyrolyzed at 1200 °C) [28]. This fact promises further optimization of the composite cathode.

**Figure 10:**
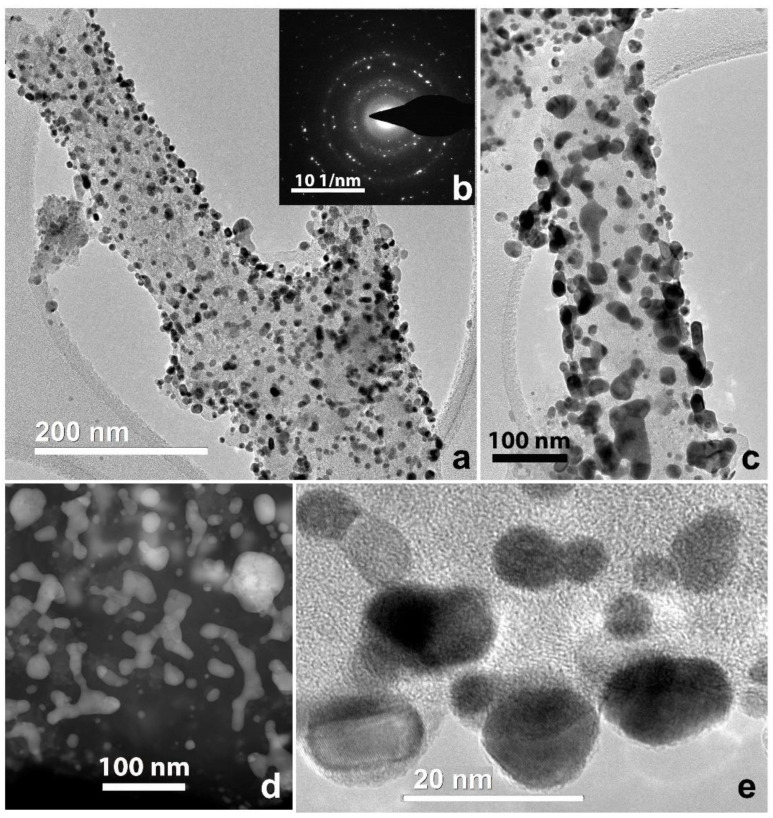
Structure of Pt/1’ (after working in MEA): (**a**) general view and (**b**) corresponding SAED pattern; (**c**) changed morphology of Pt crystals; (**d**) HAADF STEM images of polycrystalline particles; (**e**) HRTEM image of particles.

Platinum morphology in CNF/Zr/Ni/Gd-based composite cathode significantly changed after working in the fuel cell (sample Pt/1′). For Pt/1′, particle sintering was observed as a result of long interactions with *o*-phosphoric acid at 180 °C and electrochemical cathodic reactions on platinum surface (Figure 10).

**Figure 11:**
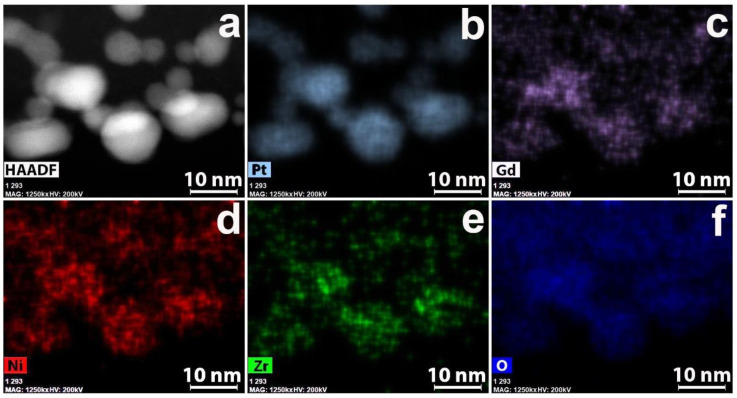
(**a**) HAADF STEM, and EDX distribution maps for (**b**) Pt, (**c**) Gd, (**d**) Ni, (**e**) Zr and (**f**) O for Pt/1’ after working in membrane-electrode assembly.

For some nanofibers a crust of nanoparticles was formed. Both platinum conglomerates and residual small size spherical platinum particles of >2 nm were observed on nanofibers. Needle-type platinum morphology was absent. Z-contrast STEM images (Figure 10d) of the particles allow to distinguish their features. The majority of particles represented polycrystals of random shape with rounded outlines. Twins and crystallites of rounded and elongated shape were present. The nanofiber structure was quite curly with high porosity. Graphene layer sets were observed in carbon material. HAADF STEM and EDX distribution maps are shown in Figure 11.

As it can be seen from Figure 11, after sample performance in MEA of a fuel cell, a substantial redistribution of Zr, Ni, and Gd occurs and their locations coincide with Pt. All experiments for different nanofiber composite materials expose similar results and can be explained by formation of metal phosphates.

### 3.3. CNF/Co/Sm

**Figure 12:**
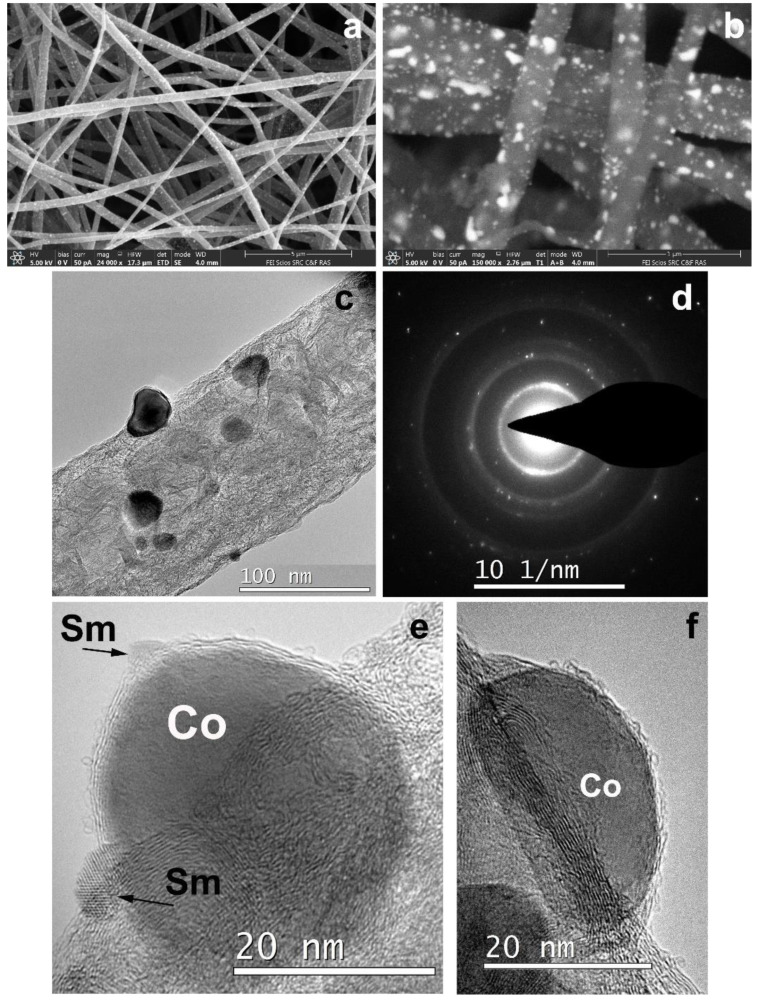
Structure of 3: (**a**) and (**b**) SEM images of CNF containing metal particles; (**c**) enlarged image of carbonized nanofiber; (**d**) Sm and Co reflexes in SAED pattern; (**e**) and (**f**) HRTEM images of Sm and Co particles grown on the surface of CNF.

In order to obtain CNF/Co/Sm composite 3, cobalt acetate and samarium chloride salts were added to a solution of polyacrylonitrile in DMF for electrospinning. After electrospinning the nanofiber mat was pyrolyzed at 1200 °C. This temperature was chosen to try a full reduction of the reduced metals. The obtained nanofibers containing thus formed metal nanoparticles are shown in Figure 12.

The SEM images (Figure 12a,b) indicated a significant number of particles that differed in shape and size and were distributed quite uniformly. The largest ones possibly reached 150–200 nm. Bright field (BF) TEM image of a CNF part (Figure 12c) revealed very high crystallinity degree in CNF after high temperature pyrolysis. Besides, as it was already described in our previous studies [29], Fe and Ni particles catalyze the growth of graphene layers and promote higher fiber porosity and inner channel formation. According to the HRTEM, Co played the same role in crystallinity increase.

**Figure 13:**
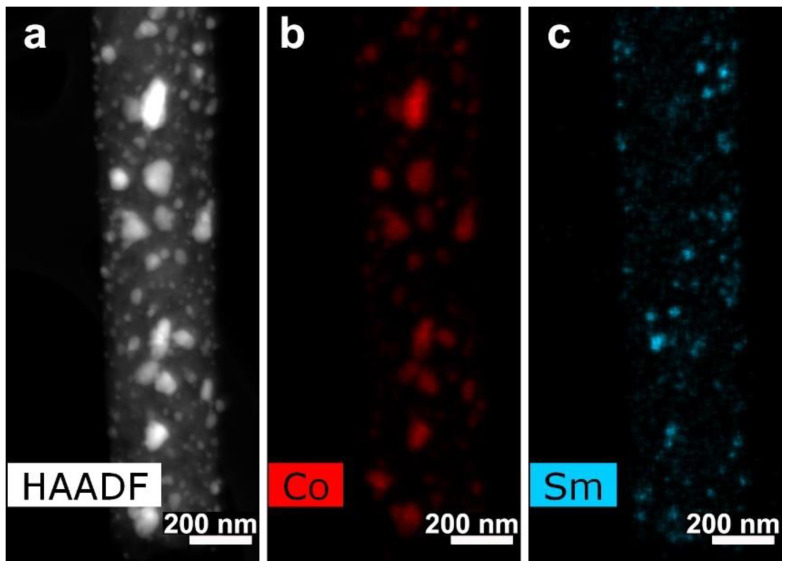
(**a**) HAADF STEM and EDX distribution maps for (**b**) Co and (**c**) Sm for sample 3.

The HRTEM images (Figure 12e,f) revealed isolate particles surrounded by graphene layers on the CNF surface. The specifics of this sample were the presence of smaller-sized particles (a few nanometers) on various surfaces wrapping Co particles (dozens of nanometers). Interplane distance analysis according to HRTEM indicated that large particles were those of Co and smaller particles were those of Sm with a rhombohedral/hexagonal lattice. The composite crystal structure study was carried out by the selected area electron diffraction (SAED) pattern analysis. Single reflections on SAED correspond to interplanar distances d_hkl_ = 3.06, 2.93, 2.63, 2.14 Å, indicating Sm in a form of rhombohedral lattice. Interplanar distances d_hkl_ = 2.15, 2.02, 1.89 Å correspond to Co hexagonal lattice phase. However, the Co crystal lattice structure is in question so far and requires additional investigations because our previous Co samples crystallized in a form of face-centered cubic lattice. HAADF STEM and EDX distribution maps of Sm and Co are shown in Figure 13.

**Figure 14:**
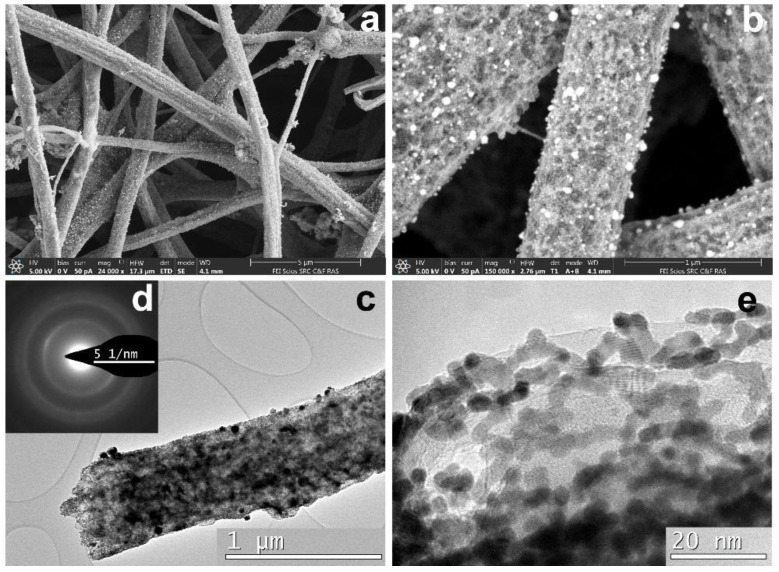
CNF/Co/Sm structure for Pt/3: (**a**) and (**b**) SEM images; (**c**) and (**d**) a fragment of CNF and corresponding SAED pattern; (**e**) Pt morphology.

As it is seen from Figure 13, samarium and cobalt were placed separately, which means they probably did not form a compound such as SmCo_5_**.** Sm is dispersed more smoothly than Co. Platination of the CNF/Co/Sm composite leads to Pt nanoparticles formation on the nanofibers according to SEM (Figure 14).

**Figure 15:**
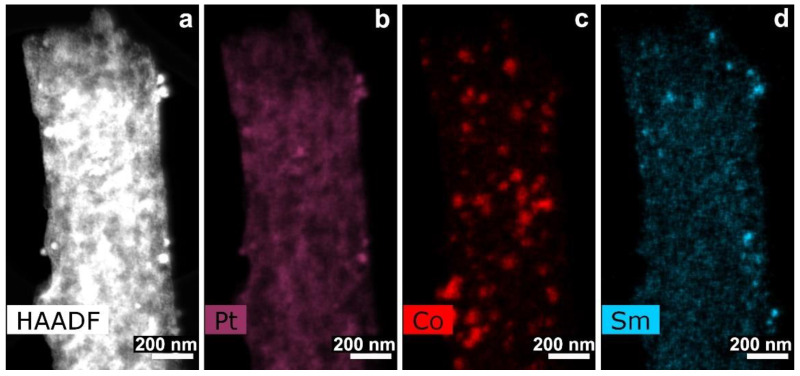
(**a**) HAADF STEM image and EDX distribution maps for (**b**) Pt, (**c**) Co and (**d**) Sm for sample Pt/3.

In SEM images taken at intermediate magnifications some “blobs” and Pt outgrowths with sponge structure are found (Figure 14a,b). At higher magnification the nanofibers look like bundles, their structure being different from that of other samples. The nanofiber surface is covered by small nanometric Pt nanoparticles of spherical shape (Figure 14c,e). Such Pt morphology on carbon composite nanofibers was observed for the first time. TEM images (Figure 14) revealed differences in platinum morphology. According to BF TEM images and selected area electron diffraction (SAED) patterns all fibers were uniformly covered by Pt nanoparticle layers of a few nanometers in size (<10 nm). Nanoparticles of predominantly spherical shape of 2–3 nm were observed. Polycrystal intergrowths formed by a few nanoparticles were also detected. Such Pt nanoparticle morphology and small Pt nanoparticle size can be explained by the fact that preliminary deposited Co nanoparticles promoted higher CNF crystallinity degree. The details of the process were discussed in Reference [29], where it was shown that Fe particles served as a catalyst moving inside the volume of carbon fibers, making channels covered with crystalline graphene layers providing a remarkable contribution to their crystallinity. The channels displayed the structure as multi-walled carbon nanotubes during their catalytic growth on Fe, Ni or Co nanoparticles [54,55,56,57,58]. The same effects of crystallization of CNFs at heat treatment were observed in the case of Ni and as we supposed for Co particles. Such CNF structure favors the increase in the number of platinum nucleation centers and, from the other side, promotes the increase in fiber porosity and results in isolation of the nucleation centers. So that, particles and clusters obtained during platination appear to be separated and prevent their coagulation (size growth). Corresponding EDX distribution maps are shown in Figure 15.

It is clearly seen that Co and Sm were situated in different locations in the same way as it was found before platinization. The polarization curve for the platinated CNF/Co/Sm composite material Pt/3 used as cathode in MEA at 180 °C with PBI-OPht membrane is shown in Figure S3. This type of Co and Sm containing nanofiber material requires further optimization of pyrolysis temperatures. It is necessary to emphasize that electrochemically active surface area of Pt for composite material Pt/3 was 30.3 m^2^ g^−1^ (Figure S2), which is sufficient for using it as a cathode in MEA. Further optimization of such type a cathode is needed.

### 3.4. CNF/Zr/Ni/Ce

The sample of PAN/Vulcan/Zr/Ni/Ce composite material 4 was obtained by the method of electrospinning from a solution of PAN with added Ni, Zr, and Ce salts, followed by further oxidation (350 °C, air) and pyrolysis (1000 °C, vacuum). The material contains 5.1% Ni, 0.92% Zr, and 0.85% Ce according to elemental analysis. The electrical conductivity of this material reached 12.3 S cm^−1^ which makes it sufficient for use in fuel cell electrodes as it was confirmed by our previous studies [31,32,33].

**Figure 16:**
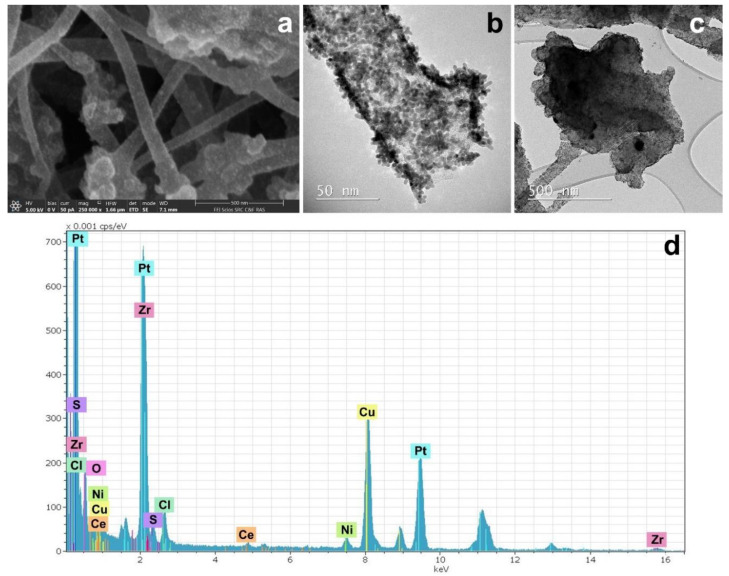
Structure of Pt/4 composite material pyrolyzed at 1000 °C: (**a**) SEM image; (**b**) and (**c**) TEM images of single fiber and fragment of the carbon black; (**d**) the EDX spectrum.

A SEM image of the composite material Pt/4 (Figure 16) shows that fibers and carbon black particles were covered by a platinum layer quite uniformly. The Pt nanoparticle morphology was diverse including both spherical particles of 2–5 nm and needles up to 20 nm in length (Figure 16b). A lot of carbon black particles densely covered by Pt nanoparticles are visible in the sample (Figure 16c). As it was already shown for the previous sample, distribution, size, and morphology of the metal (Ni and Zr) particles were not changed. Cerium was present in the sample as small particles of a few nanometers in size.

### 3.5. XPS Studies

#### 3.5.1. General

During chemical analysis of multicomponent systems one of the main problems is charge referencing because it influences an adequate determination of chemical shifts. Taking this into account, a curve fitting procedure was used. Almost all the C 1s spectra demonstrated a π-π* plasmon, that is why a fingerprint of sp^2^-state, the C 1s spectrum of highly oriented pyrolytic graphite (HOPG) as main element of a basic set, was used.

The parameters of spectrum were modified for the best description of the low-energy part of the spectrum by convolution with the Gauss profile to account for sample inhomogeneity and normalization of signal intensity in π-π* plasmon region. The relative area of highly oriented pyrolytic graphite (HOPG) spectrum was used as a measure of sp^2^-hybridization degree. Binding energies of another Gaussian profiles were chosen, according to reported chemical shifts [59,60]. Binding energies of ~282.0, ~283.1, 284.44, ~284.8, ~285.6, ~286.7, ~287.0, ~288.1, and 289.4 eV were associated with C–C/C–H group related to edge defects and low molar mass species [61], carbon-metal bonds, sp^2^-state, sp^3^-state, C–N, C–OH/C–O–C, O–C–O, C(O)N and C(O)O groups, respectively. The fitting parameters are presented in Table S1.

#### 3.5.2. Samples 3 and Pt/3

**Figure 17:**
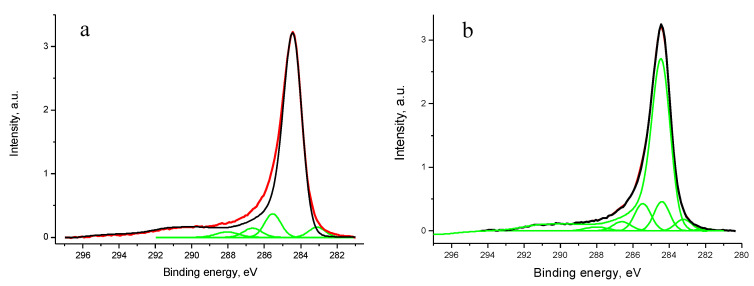
The C 1s photoelectron spectra of samples 3 (**a**) and Pt/3 (**b**).

No peaks of contaminations were recorded in the survey spectra of the examined samples. According to XPS quantitative analysis the compositions of samples 3 and Pt/3 are C_94.7_N_0.6_O_5.0_Co_0.1_Sm_0.3_ and C_86.7_N_1.6_O_5.0_Pt_6.2_Co_0.04_Sm_0.4_. Figure 17 shows the C 1 s spectra of samples 3 and Pt/3, relative intensities of sp^2^-state in samples 3 and Pt/3 are 0.86 and 0.75, respectively.

After Pt deposition, small changes in the C 1s were observed. They were related to decrease in relative intensity of sp^2^–state from 0.86 to 0.75 and an increase in sp^3^-carbon. Figure S4 shows the dependence of signal intensity in the binding energy region characteristic of Co 2p spectrum. The vertical lines mark the positions of Co 2p_3/2_ and Co 2p_1/2_ levels of CoO. A weak signal is observed in Co 2p_3/2_ peak region but no related signal in the region of Co 2p_1/2_. Therefore, it is difficult to identify presence of Co within information depth of XPS. In the abovementioned compositions of samples of 3 and Pt/3 the Co contents are maximum possible evaluating data.

**Figure 18:**
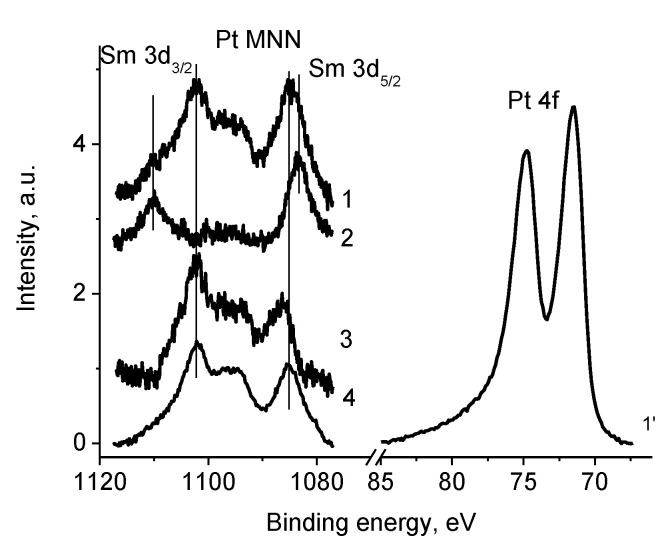
The Sm 3d photoelectron spectra of samples Pt/3 (1), 3 (2), their difference spectrum (3), Pt MNN Auger spectrum (4) and Pt 4f_7/2_ (1’).

Although according to the preparation conditions Co_5_Sm must have been produced, the Sm 3d spectra were measured because the Sm 3d atomic sensitivity factor was approximately five times more than that of Co 2p (Figure 18). The Sm 3d_5/2_ and Sm 3d_3/2_ peaked at 1083.3 eV and 1110.3 eV corresponded to Sm_2_O_3_ [62].

Figure 5 shows that the Sm 3d spectrum of sample Pt/3 differs essentially from that of sample 3. It is caused by overlapping with Pt MNN Auger spectrum from Pt nanoparticles. Thus, the spectrum of sample Pt/3 in this energy region can be described as superposition of Sm 3d photoelectron spectrum of Sm_2_O_3_ and Pt MNN Auger spectrum of Pt^0^ state. This fact is supported by Pt 4f spectrum presented in Figure 5. The binding energies of the Pt 4f_7/2_ and Pt 4f_5/2_ are 71.5 and 74.8 eV. Both the binding energy and the Pt 4f_5/2_–4f_7/2_ spin-orbit splitting of 3.27 eV indicate that Pt is in the zero-oxidation state. The energy shift of 0.38 eV relative to the spectrum of Pt foil [46] may be assigned to the size effect in photoelectron spectra [63]. Both the binding energy and the shift indicate that nanoparticle size was less than 5 nm; that is in accordance with TEM data. The metallic line shape with an asymmetric smooth high-energy tail resulting from photoelectrons scattering on electrons of conduction band indicates metallic properties of Pt nanoparticles as well. Pt deposition induces increase in N content by 2.7 times and change in relative intensities of peaks at 400.9 and 398.7 eV [46,62]. Intensity ratios of these peaks for samples 3 and Pt/3 are 1.24 and 0.55, respectively. Binding energy of 398.7 eV, as a rule, is assigned to bonds between metal and nitrogen atoms and may relate to both Co–N and Pt–N bonds. It should be noted that increase in nitrogen content may be caused by diffusion induced by Pt atoms.

**Figure 19:**
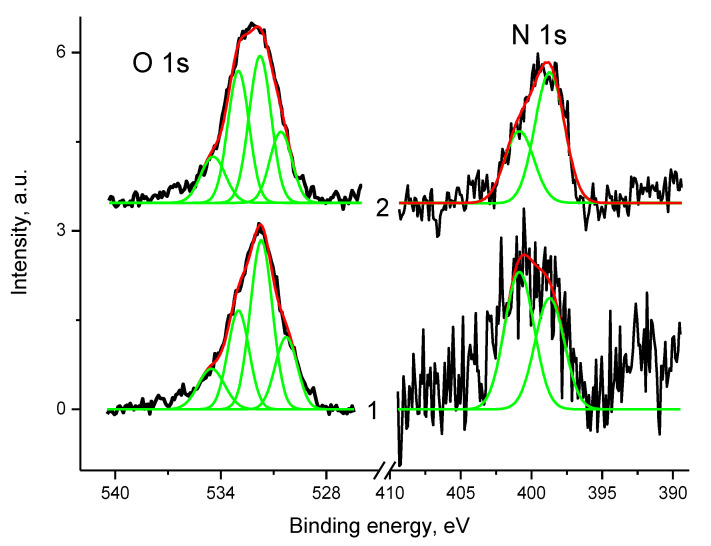
The O 1s and N 1s photoelectron spectra of samples 3 (1) and Pt/3 (2) (initial spectra – black, basic curves – green, resulting curves – red).

A detailed analysis of the O 1s in Figure 19 corresponds to that of the C 1s spectra in Figure 17. Due to low metal contents and Pt in Pt^0^ state the O 1s spectra are mainly determined by CO groups.

The spectrum of sample 3 was fitted with four peaks at 530.3, 531.7, 533.0, and 534.5 eV with Gaussian widths 1.25, 1.25, 1.2, and 1.4 eV and relative intensities 0.19, 0.44, 0.25 and 0.13, respectively. The spectrum of sample Pt/3 was fitted with four peaks at 530.6, 531.8, 533.0, and 534.4 eV with Gaussian widths 1.25, 1.25, 1.2, and 1.5 eV and relative intensities 0.18, 0.37, 0.32. and 0.13, respectively. The peaks were associated with chemisorbed water, C(O)N and epoxy groups, and physadsorbed water. It appeared that water conservation results from highly porous morphology of the sample despite spectra recording in ultrahigh vacuum.

Thus, the XPS results suggest that Sm atoms are in Sm^3+^ state. Cobalt atoms are positioned below XPS sampling depth.

#### 3.5.3. Samples 4 and Pt/4

According to XPS quantitative analysis the compositions of samples 4 and Pt/4 are C_90.6_N_1.4_O_7.4_Ni_0.3_Ce_0.2_Zr_0.3_ and C_38.0_N_8.2_Pt_45.8_O_8.0_. Relative intensities of sp^2^-state in samples 4 and Pt/4 are 0.71 and 0.59, respectively (Figure S5).

**Figure 20:**
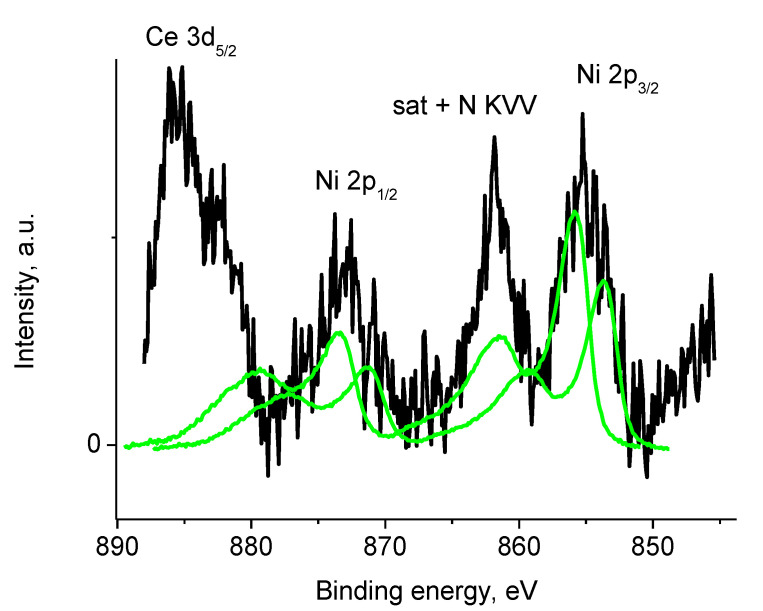
Photoelectron spectrum of sample 1 in the energy interval of Ni 2p spectrum (initial spectrum—black, Ni 2p spectra—green).

The binding energies of Zr 3d_5/2_ and Zr 3d_3/2_ peaks at 182.4 and 184.8 eV and Zr 3d_5/2_/Zr 3d_3/2_ branching ratio of 1.5 indicate only one chemical state Zr^4+^. Figure S6 shows that Zr was not detected within the XPS sampling depth of sample Pt/4, while the P 2s peak at ~189.9 eV associated to phosphate was recorded. No peak characteristics of Ni were recorded in the spectra of sample Pt/4. Figure 20 displays energy interval characteristic of Ni 2p spectrum of sample 4 which demonstrates overlapping Ce 3d_5/2_, Ni 2p, and N KVV spectra. The peak position of Ni 2p_3/2_ and 2p_1/2_ were observed at 855.0 and 872.7 eV, a satellite of the Ni 2p_3/2_ peak overlapped with N KVV Auger spectrum was observed at 861.7 eV, while that of Ni 2p_1/2_ overlapped with the Ce 3d_5/2_ peak.

Taking into consideration the Ni 2p_3/2_ peak width, the Ni 2p spectrum was fitted with two reference spectra associated with two chemically different Ni entities as shown in Figure 20: NiO and Ni(OH)_2_ with Ni 2p_3/2_ binding energies of 853.6 and 855.8 eV, respectively [64,65,66].

The NiO/Ni(OH)_2_ intensity ratio was 2/3. The Ni 2p spectrum of Ni(OH)_2_ was used as a reference spectrum of Ni^2+^ state. However, it should be noted, that such an interpretation is rather approximate, and the number of Ni entities may be higher.

Figure S7 displays energy region of Ce 3d spectra. It clearly shows the absence of Ce 3d signal in the spectrum of Pt/4 and presence of N KVV Auger spectrum. The peak at ~916.4 eV in the Ce 3d spectrum is characteristic of Ce^4+^ state and those at 885.8 and 904.0 are characteristic of Ce^3+^ state. Their relative intensities were 0.15 and 0.85, respectively. The O 1s photoelectron spectra of samples 4 and Pt/4 were very close and coincided with that of sample 3.

Two ways of fitting the N 1s spectrum of sample Pt/4 with two and three peaks were used (Figure S8). In the first case, Gaussian widths exceeded essentially those of the C 1s peaks; the binding energies of 398.1 and 399.9 eV and Gaussian width of 1.35 eV were obtained. In the second case the corresponding values were 398.0, 399.5 and 400.8 eV and 1.2 eV. In this case a peak width was a measure of homogeneity. The relative intensities of the peaks were 0.47, 0.37, and 0.16. Taking into account the sample composition, the peaks at 398.0, 399.5, and 400.8 eV at 398.0 eV were assigned to Pt–N bonds, non-completely pyrolyzed PAN and C(O)N groups.

Similar approaches were used for the curve fitting procedure of the N 1s spectrum of sample 4 but with three and four peaks as shown in Figure S9. It should be noted, that in this case a binding energy of low-energy peak of 398.6 eV was 0.6 eV more than in previous case. Nevertheless, it should be associated with metal-nitrogen bond and may be assigned to Ni–N, Ce–N and Zr–N bonds. However, it is very difficult to specify them because the metals are mainly in oxide form. The peaks at 400.16, 401.69 and 407.52 eV are assigned to C(O)N, NH_3_^+^ and NO_3_ groups, respectively [59]. The relative intensities of four peaks are 0.33, 0.30, 0.17, and 0.20.

Figure S10 shows the Pt 4f spectrum of sample Pt/4. The binding energies of the Pt 4f_7/2_ and Pt 4f_5/2_ are 71.5 and 74.8 eV. The values are the same as for sample Pt/3 and the line shapes are similar as well. Therefore, Pt is in the zero-oxidation state and nanoparticle size is less than 5 nm.

Thus, XPS data show evidence of Ce^3+^ and Ce^4+^ states.

#### 3.5.4. Samples 1 and Pt/1

According to XPS quantitative analysis the compositions of samples 1 and Pt/1 are C_81.1_N_2.0_O_13.7_Ni_0.4_Zr_0.3_Gd_2.7_ and C_77.1_N_3.8_O_6.6_Ni_0.6_Pt_11.5_Zr_0.1_Gd_0.3_. The C 1s photoelectron spectrum of sample 1 is similar to that of sample Pt/1 (Figure S11). No changes were observed after Pt deposition. The relative intensity of sp^2^-state in both samples is 0.57, while the portions of sp^3^-defects are slightly different, namely 0.23 and 0.24, respectively.

In the Ni 2p_3/2_ spectrum of samples 1 and Pt/1 (Figure S12) two peaks at 852.7 and 855.1 eV associated with Ni^0^ and Ni^2+^ states were deconvoluted. The Ni^0^/Ni^2+^ intensity ratios are 0.38 and 0.39, respectively. Binding energy of 855.2 eV corresponds to state Ni_2_O_3_⋅H_2_O [67]. The Ni 2p spectra of Ni foil and sample Ni(OH)_2_ were used as reference spectra of Ni^0^ and Ni^2+^ states.

**Figure 21:**
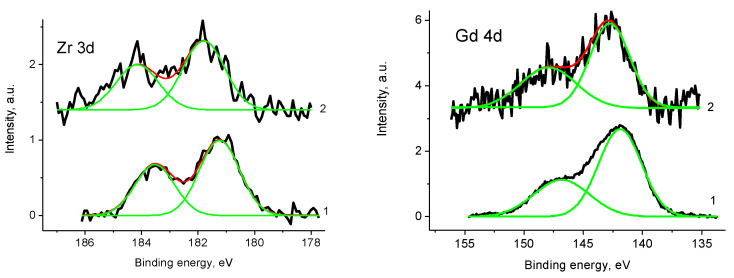
The Zr 3d and Gd 4d Photoelectron spectra samples 1 (1) and Pt/1 (2) (initial spectra—black, basic curves—green, resulting curves—red).

It should be mentioned that Pt deposition induced positive shift Zr 3d, Gd 4d and O 1s spectra and appearance of well resolved peak at 397.8 eV in the N 1s spectrum assigned above to Pt-N bonds, and peak at 407.0 eV assigned to NO_3_ group. The Zr 3d_5/2_ and Gd 4d_5/2_ peaks of both samples correspond to Zr^4+^ and Gd^3+^ states, but not a higher charged state on the metal atoms. The observed energy shift is caused by formation of a new phase with lower electroconductivity and manifestation of differential charging. Figure 21 displays the Zr 3d and Gd 4d photoelectron spectra of samples 1 and Pt/1. The binding energies of the Zr 3d_5/2_ and 3d_3/2_ of samples 1 and Pt/1 are 181.2/183.5 eV and 181.8/184.1 eV, respectively. The binding energies of the Gd 4d_5/2_ and 4d_3/2_ are 142.8/147.9 and 141.9/146.8 eV.

**Figure 22:**
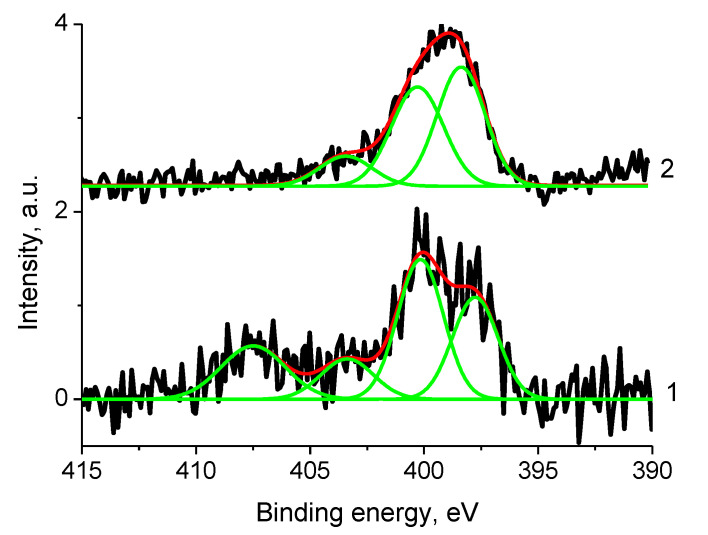
The N 1s photoelectron spectra of samples 1 (1) and Pt/1 (2) (initial spectra – black, basic curves – green, resulting curves – red).

Figure 22 displays the N 1s photoelectron spectra of samples 1 and Pt/1 fitted with four and three peaks at binding energies of 397.8, 400.16, 403.4, 407.5 and 398.4, 400.3, 403.4 with relative intensities of 0.28, 0.39, 0.13, 0.21 and 0.46, 0.41, 0.13, respectively.

The O 1s line shape of samples Pt/1 and 1 coincides with that of sample 3. However, the O 1s spectrum of sample 1 is shifted to low energy region by 1 eV due to differential charging. Figure S13 shows the Pt 4f spectrum of sample Pt/1. The binding energies of the Pt 4f_7/2_ - Pt 4f_5/2_ spin-orbit doublet is 71.3 and 74.6 eV. The Pt 4f_7/2_ core level binding energy shift by 0.2 eV and asymmetric high energy tail evidence on Pt^0^ state and nanoparticle size less than 5 nm.

#### 3.5.5. Sample Pt/1′

The XPS quantification data of sample **Pt/1’** determined with Mg and Al anodes are C_61.9_N_2.0_O_20.1_Ni_0.1_Pt_4.5_Zr_0.2_Gd_2.5_F_0.1_P_7.6_ and C_59.7_N_2.2_O_24.2_Ni_0.1_Pt_3.0_Zr_0.2_Gd_2.9_F_0.1_P_7.1_. They evidence inhomogeneous distributions of elements in the vicinity of composite surface. The C 1s spectrum presented in Figure S14 and corresponding parameters presented in Table 1 indicate that the relative intensity of carbon atoms in sp^2^-state is 0.48 and ~0.25 where carbon atoms form C–O bonds. The deconvoluted peak at 285.07 eV with relative intensity of 0.22 is assigned to sp^3^-carbon and possible defects in graphite-like structure.

**Figure 23:**
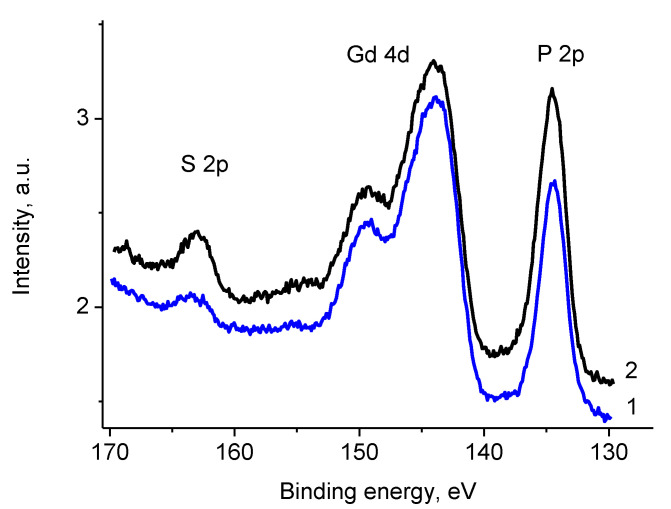
The S 2p, Gd 4d, and P 2p photoelectron spectra of sample Pt/1′ recorded using Al Kα (1) and Mg Kα (2) radiations.

Figure 23 clearly shows surface enrichment with sulfur and phosphorus relative to gadolinium. P 2p binding energy of 134.5 eV characterizes phosphates and polyphosphates. The Gd 4d_5/2_ and 4d_3/2_ binding energies of 144.1 and 149.6 eV and Gd 3d_5/2_ and 3d_3/2_ binding energies of 1186.5 and 1220.6 eV (Figure S15) are associated with both oxide and phosphate. The S 2p binding energy of 163.4 eV corresponds to C–S bonds [59] formed with aromatic or aliphatic carbons.

**Figure 24:**
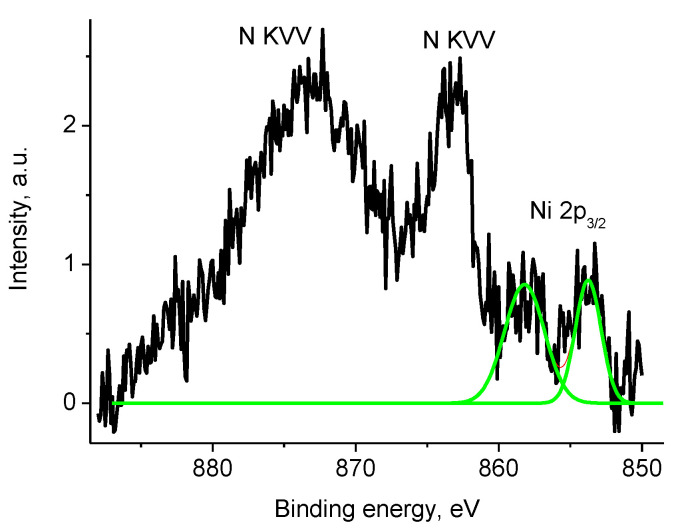
The Ni 2p photoelectron spectrum of sample Pt/1’ (initial spectra—black, basic curves—green, resulting curve—red).

As in previous Pt containing samples, the metallic line shape and Pt 4f_7/2_ and 4f_5/2_ binding energies of 71.7 and 75.1 eV indicate that chemical state of Pt atoms is Pt^0^ and the size of nanoparticles is smaller than 4 nm (Figure S15). Figure 24 displays the Ni 2p photoelectron spectrum of sample Pt/1′ overlapped with the N KVV Auger spectra. The Ni 2p_3/2_ spectrum was approximated by two Gaussian functions with binding energies of 853.8 and 858.2 eV.

The value of 853.8 eV was intermediate between Ni metal (852.7 eV) and NiO (854.3 eV). The satellite at 858.2 eV indicates that Ni was in the oxide form. These data may be explained by presence of two Ni states, namely, Ni^0^ and Ni^2+^. It is very difficult to distinguish between them; that is why no attempt was made to separate Ni^0^ and Ni^2+^ contributions.

**Figure 25:**
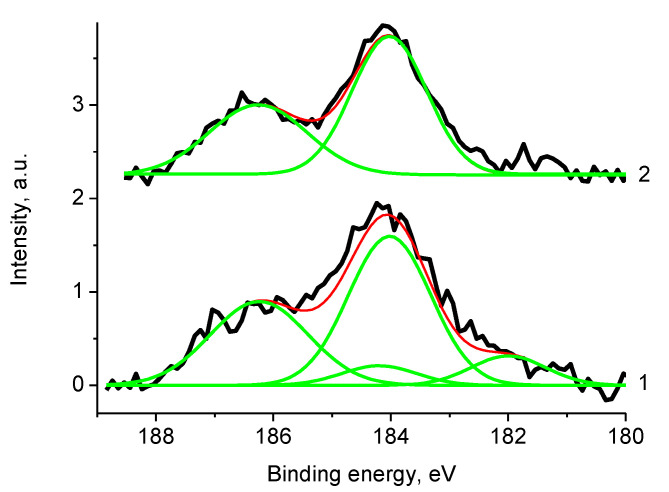
The Zr 3d photoelectron spectra of sample Pt/1′ measured with Mg (1) and Al (2) anodes (initial spectra—black, basic curves—green, resulting curves—red).

Figure 25 displays the Zr 3d photoelectron spectra of sample Pt/1′ measured with Mg and Al anodes. Binding energies of the Zr 3d_5/2_ and Zr 3d_3/2_ peaks at 184.0 and 186.2 eV measured using Al Kα radiation, and Zr 3d_5/2_/Zr 3d_3/2_ branching ratio of 1.5 characterize zirconium phosphate. Both zirconium phosphate and oxide were recorded with Mg Kα radiation. Binding energies of the Zr 3d_5/2_ and Zr 3d_3/2_ peaks at 182.0 and 184.2 eV correspond to zirconium oxide. Mg Kα radiation provides more surface sensitive mode and results from the fact that zirconium oxide is in top layers.

#### 3.5.6. Sample 2

**Figure 26:**
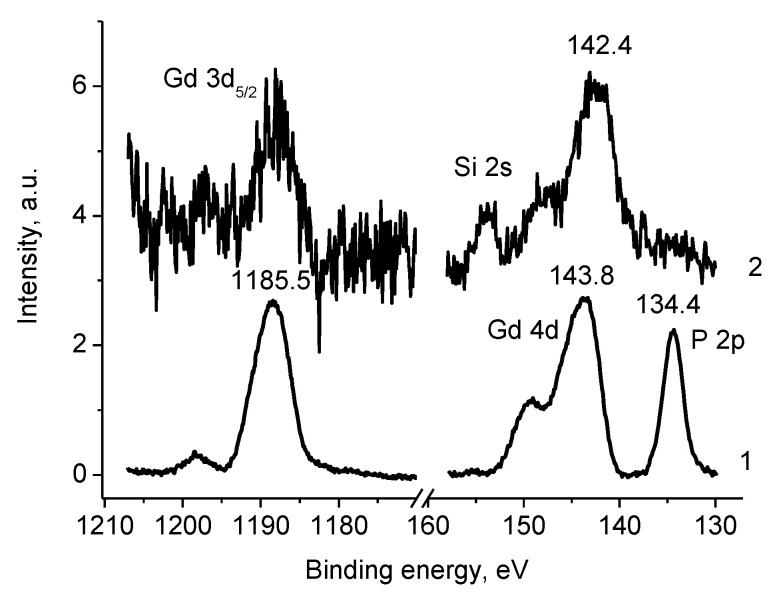
The Gd 3d photoelectron spectra of samples Pt/1′ (1) and 2 (2).

XPS quantification data of sample 2 determined with Mg and Al anodes are C_87.7_N_0.6_O_7.0_Ni_0.3_Zr_0.07_Gd_0.03_ and C_77.7_N_1.2_O_14.6_Ni_0.3_Zr_0.03_Gd_0.1_. The C 1 s spectrum does not show (Figure S16) satellite, related to π-π* plasmon. The relative intensity of C-O bonds is 0.46. Figure 26 presents a comparison of the Gd 4d and Gd 3d_5/2_ core level spectra of samples Pt/1′ (1) and 2 (2). The differences in the peak positions clearly prove that Gd in sample 2 is in oxide form, while that in sample Pt/1′ Gd is mainly in phosphate form.

**Figure 27:**
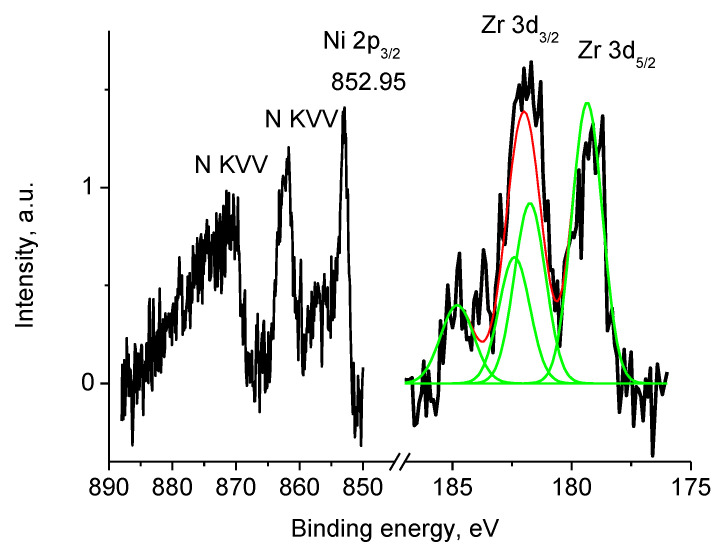
The Ni 2p and Zr 3d photoelectron spectra and N KVV Auger spectra of sample 2 (initial spectra—black, basic curves—green, resulting curve—red).

The Zr 3d spectrum in Figure 27 was fitted with two spin-orbit doublets at binding energies of 179.4/181.8 and 182.4/184.8 eV with relative intensities 0.69 and 0.31, which may be assigned to Zr^+^ and Zr^4+^ states.

**Figure 28:**
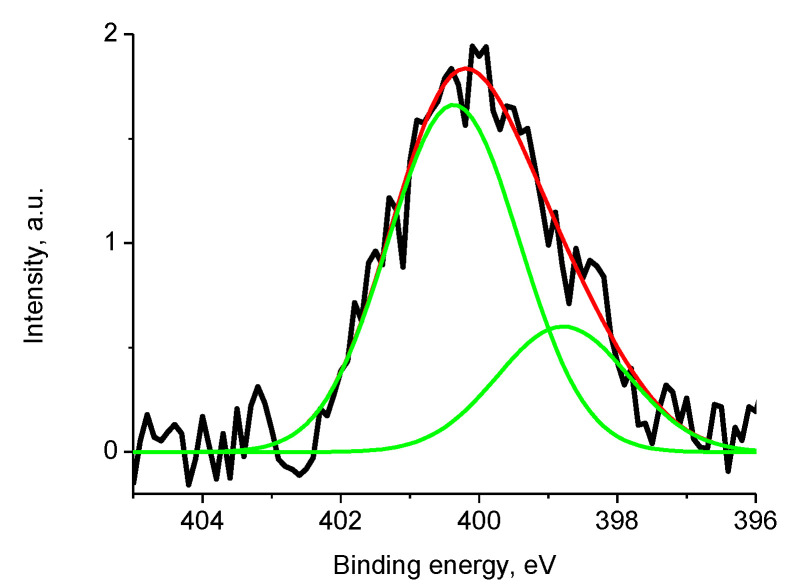
The N 1s photoelectron spectrum of sample 2 (initial spectra—black, basic curves—green, resulting curve—red).

The Ni 2p_3/2_ binding energy of 853.0 eV is associated with Ni^0^. The energy shift of ~0.3 eV was caused by size effect and its value indicates that nanoparticle size is less than 5 nm. The N 1s spectrum (Figure 28) was fitted with two peaks at 398.8 and 400.4 eV with relative intensities of 0.27 and 0.73 assigned to metal-nitrogen bonds and OC(O)N groups.

In summary, it is important to mention that Pt, deposited on CNF composite material, is mainly in the Pt^0^ state, according to XPS analysis.

## 4. Conclusions

Polyacrylonitrile is one of the most important polymers demonstrating unique innate ability to be transformed into carbon materials, especially carbon fibers. In the last two decades, special attention has been paid to electrospun nanofiber mats based on PAN which are very promising precursors to obtain carbon nanofiber materials for electrochemical devices, for instance, highly durable gas-diffusion electrodes of HT-PEMFC.

Such electrocatalytic systems of higher performance, such as Pt–M and Pt–MOx (on carbon black support) characterized by better performance, were introduced into CNF self-supporting mats which are more durable materials used in HT-PEM fuel cell GDEs improving their durability. Higher performance of the above GDE (when the support was pyrolyzed at 1200 °C) was observed at high current densities (>1.2 A cm^−2^) compared to commercial Celtec^®^P1000 electrode. This fact can be explained by decreasing of mass transfer limitations in highly porous CNF-based electrodes compared to commercial carbon black ones which exhibit “flooding” at high current densities due to the considerable water formation under HT-PEM fuel cell operating conditions.

In this work we developed our concept of new GDE design based on platinated freestanding CNF mats obtained by PAN pyrolysis. In order to improve cathode catalytic properties in ORR reaction we have synthesized, for the first time, new composite CNF materials containing transition metals and lanthanides as the support for Pt electrocatalyst and material probing as cathodes of HT-PEMFC based on polybenzimidazole PBI–OPht membrane.

For CNF/Zr/Ni/Gd, annealed at 1000 °C under vacuum (sample 1), additional annealing at 1200 °C (sample 2) changes morphology of the composite and deposited Pt. Finally, it affects polarization curve data of HT-PEMFC. In general, the performance is lower at low current density, but it becomes higher at high current densities (>1.2 A cm^−2^) compared to sample Pt/1 and commercial cathode. An attempt to exchange Zr, Ni and Gd to Sm and Co saving pyrolysis temperature of 1200 °C (sample Pt/3) does not reveal the same benefit of higher performance. Probably, it is related to the properties of the added metals rather than to pyrolysis temperature, so that pyrolysis conditions must be optimized. An exchange of Gd in sample Pt/1 to Ce (sample Pt/4) led to lower performance compared to Pt/1. The compatibility as well as stability of transition metal salts and lanthanide salts with polyacrylonitrile in dimethylformamide dopes was proved, and the open surface electrospinning method (Nanospider^®^ technology) allowed us to obtain non-woven composite nanofiber mat precursors. After their oxidation and pyrolysis, new composite CNF supports, as follows, CNF/Co/Sm, CNF/Zr/Ni/Gd, and CNF/Zr/Ni/Ce were prepared for Pt catalyst deposition. High-resolution electron microscopy confirmed that inorganic additives were uniformly distributed in the matrix of pyrolyzed polyacrylonitrile. The detailed look into lanthanide and transition metal oxidation states was performed by XPS. The collected data revealed that Pt deposited on CNF composite material was mainly in the Pt^0^ state; however, it formed Pt–N bonds on the surface of pyrolyzed PAN. Platinum deposition stimulated increased N content in the vicinity of the Pt surface region. Chemical states of other atoms differed from sample to sample, namely, Ni atoms formed Ni^0^ and Ni^2+^ states; Gd atoms were in Gd^3+^ state as oxide; Zr atoms were in Zr^2+^ state as oxides. For Ce-containing sample, the XPS data evidenced that cerium was both in the Ce^3+^ and Ce^4+^ states. Data for Sm-containing samples proved that its atoms were in the Sm^3+^ state only. The C 1s XPS spectra showed that carbon atoms of the CNF surface were mainly in the sp^2^-state. Some carbon atoms were in the sp^3^ “defect states”, while others formed different bonds with oxygen and nitrogen. For Ni-containing samples, annealed at 1200 °C, only Ni^0^ was found, whereas 1000 °C pyrolysis yielded Ni^0^ and Ni^2+^ states.

It is very important to note that metal phosphate species in all investigated samples appeared on the surface of nanofibers after testing composite materials as cathodes in fuel cell. Pt ECSA values obtained by cyclic voltammetry are in agreement with high performance of such nanocomposites in HT-PEMFC.

Three different types of Pt morphology were found according to electron microscopy. We assume that further progress in electrocatalytic systems include the development of nanocomposite synthesis methods resulting in specified Pt morphology. Nowadays, such CNF nanocomposites have been proved to be operational with good performance as “all in one” GDE in HT-PEMFC cathodes. Accumulated experience can be broadened in the field of innovative electrocatalysts, for example, alloyed Pt**_x_**Ni or Pt**_x_**Ln electrocatalysts which can be deposited on CNF with specified properties. It allows to improve the main characteristics of fuel cells with simultaneous durability increase and cost reduction of fuel cell energy units.

## Figures and Tables

**Table 1:** Elemental content and electrical conductivity for carbon nanofiber (CNF)-based samples.

Sample	%C	%N	%H	%Ni	%Zr	%Gd	%Co	%Sm	%Ce	S, S cm^−1^
1	84.73	1.75	0.91	2.1	0.8	7.3	-	-	-	13.1
2	88.25	0.55	0.27	1.4	0.7	5.3	-	-	-	23.4
3	86.27	0.61	0.23	-	-	-	6.1	3.2	-	19.2
4	87.08	1.91	0.4	5.1	0.9	-	-	-	0.85	12.3

## Supplementary Materials

The following are available online at https://www.mdpi.com/2073-4360/12/6/1340/s1, Figure S1: Polybenzimidazole PBI-OPht synthesis, Figure S2: Cyclic voltammetry for platinated samples, Figure S3: Polarization curve for sample Pt/3, Figure S4: Energy dependence of photoelectron emission in the energy region corresponding to Co 2p spectrum for the samples of 3 (1) and Pt/3 (2), Figure S5: The C 1 s photoelectron spectra of samples 4(a) and Pt/4 (b), Figure S6: The Zr 3d and P 2s photoelectron spectra of samples Pt/4 (1) and 4 (2), Figure S7: N KVV Auger spectrum of sample Pt/4 (1), photoelectron Ce 3d spectrum of sample 4 (2) and CeO_2_ (3)**,** Figure S8: The N 1s spectrum of sample Pt/4 fitted with two and three peaks, Figure S9: The N 1s spectrum of sample 4 fitted with three (1) and four (2) peaks, Figure S10: Photoelectron Pt 4f spectrum of sample Pt/4, Figure S11: The C 1s photoelectron spectra of samples 1 (1) and Pt/1 (2), Figure S12: The Ni 2p photoelectron spectra samples 1 (1), Pt/1 (2) Ni foil (3) and sample Ni(OH)_2_ (4), Figure S13: The Pt 4f photoelectron spectrum of sample Pt/1, Figure S14: The C 1s photoelectron spectrum of sample 1/Pt’, Figure S15: The photoelectron Gd 3d and Pt 4f spectra of sample Pt/1′, Figure S16: The C 1s photoelectron spectrum of sample 2, Table S1: Binding energies (BE), Gaussian widths (GW) and relative intensities (I_rel_) of some groups for the C 1s spectra of the samples investigated.
