# Supplementary material for: New Carbon Nanofiber Composite Materials Containing Lanthanides and Transition Metals Based on Electrospun Polyacrylonitrile for High Temperature Polymer Electrolyte Membrane Fuel Cell Cathodes
Source: Polymers (Basel). 2020 Jun 13;12(6):1340. doi: 10.3390/polym12061340 (PMC7362175; doi:10.3390/polym12061340)
Supplement: Supplementary file 1 [file polymers-12-01340-s001.pdf]

Supplementary information

New Carbon Nanofiber Composite Materials Containing Lanthanides and Transition Metals Based on Electrospun Polyacrylonitrile for High Temperature Polymer Electrolyte Membrane Fuel Cell Cathodes

Igor I. Ponomarev [1,*], Kirill M. Skupov [1], Olga M. Zhigalina [2], Alexander V. Naumkin [1], Alexander D. Modestov [3], Victoria G. Basu [2], Alena E. Sufiyanova [2], Dmitry Y. Razorenov [1] and Ivan I. Ponomarev [1]

[1] A. N. Nesmeyanov Institute of Organoelement Compounds of Russian Academy of Sciences, Vavilova St., 28, Moscow, 119991, Russia; kskupov@gmail.com (K.M.S.); naumkin@ineos.ac.ru (A.V.N.); razar@ineos.ac.ru (D.Y.R.); ivan.ponomarev84@gmail.com (I.I.P.)

[2] A. V. Shubnikov Institute of Crystallography of Federal Scientific Research Centre "Crystallography and Photonics" of Russian Academy of Sciences, Leninskiy Av., 59, Moscow, 119333, Russia; zhigal@crys.ras.ru (O.M.Z.); v.zhigalina@gmail.com (V.G.B); sufiyanova.alena@gmail.com (A.E.S.)

[3] A. N. Frumkin Institute of Physical Chemistry and Electrochemistry of Russian Academy of Sciences, Leninsky Av. 31, bld. 4., Moscow, 119071, Russia; amodestov@mail.ru (A.D.M.)

* Correspondence: gagapon@ineos.ac.ru; Tel.: +7-903-264-2101

PBI-OPht. Synthesis, film and membrane preparation

Polybenzimidazole PBI-OPht was obtained by our procedure [45]:

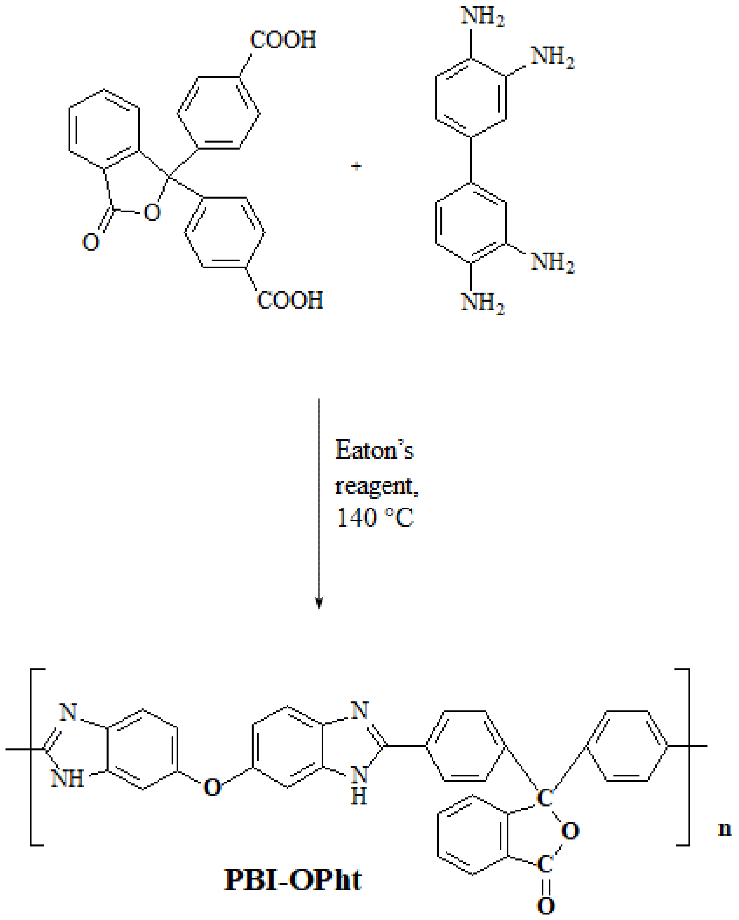

Figure S1. Polybenzimidazole PBI-OPht synthesis.

Initially 3,3',4,4'-tetraaminodiphenyl ether (0.461 g; 2 mmol) and 4,4'-diphenylphthalidedicarboxylic acid (0.749 g; 2 mmol) were mixed under dry argon flow with 3.8 mL of Eaton's reagent (P_2O_5:$MeSO_3H$ 9:1 wt/wt) in a three-neck flask equipped with a mechanical stirrer and a heater with temperature control. The mixture was stirred for 2 h at 80 °C, then for 1 h at 100 °C and for 1 h at 120 °C. Then 0.57 g (4 mmol) of P_2O_5 were added, and the reaction continued for 2 h more at 120 °C. Then the temperature was increased to 145-150 °C and the reaction continued for 2-5 h, until the dramatic increase of the mixture viscosity was observed. After that, the mixture was diluted with an equal volume of 85% H_3PO_4 and stirred to obtain homogenous solution. The latter was slowly poured into water and dispersed, then filtered, washed with water until pH 7, extracted with methanol in a Soxhlet extractor, and dried under vacuum for 5 h at 100 °C. The reduced viscosity $\eta_{red.}$= 2.1 dL/g (0.5% solution in N-MP at 25°C), which corresponds to M_w/M_n = 155000/64400 = 2.4 according to GPC [45].

Polymer films were cast from a 10% polymer solution in N-methylpyrrolidone; Zr (IV) acetyl acetonate was dissolved in N-MP and added to the polymer solution (0.01 g/1.0 g PBI) before casting on glass plates heated at 60–80 °C. After solvent evaporation (8–12 h), the films were heated in a vacuum at 160°C for 2 h for additional drying, then heated in an oven with air circulation for 1 h at 350 °C for the three-dimensional crosslinking of polymer chains [41].

The cross-linked films were doped with 77% PA at 60 °C for three days to obtain membrane materials. The resulting membrane thickness was about 50 μm. Before assembling fuel cells, membranes were stored in 85% PA at room temperature. Doping level is ~400% (~25 molecules of PA per PBI unit)

[41] Kondratenko M.S., Ponomarev I.I., Gallyamov M.O., Razorenov D.Y., Volkova Y.A., Kharitonova E.P. Khokhlov A.R. Novel composite Zr/PBI-O-Pht membranes for HT-PEFC applications. *Beilstein J. Nanotechnol.*, **2013**, *4*, 481-492.
[45] Fomenkov A.I., Blagodatskikh I.V., Ponomarev I.I., Volkova Y.A., Ponomarev I.I., Khokhlov A.R. Synthesis and molecular-mass characteristics of some cardo poly(benzimidazoles). *Polym. Sci. Ser. B*, **2009**, *51*, 166-173.

Cyclic voltammetry

Platinum electrochemically active surface area was determined by cyclic voltammetry, it is in agreement to the application of the materials as cathodes in HT-PEMFC.

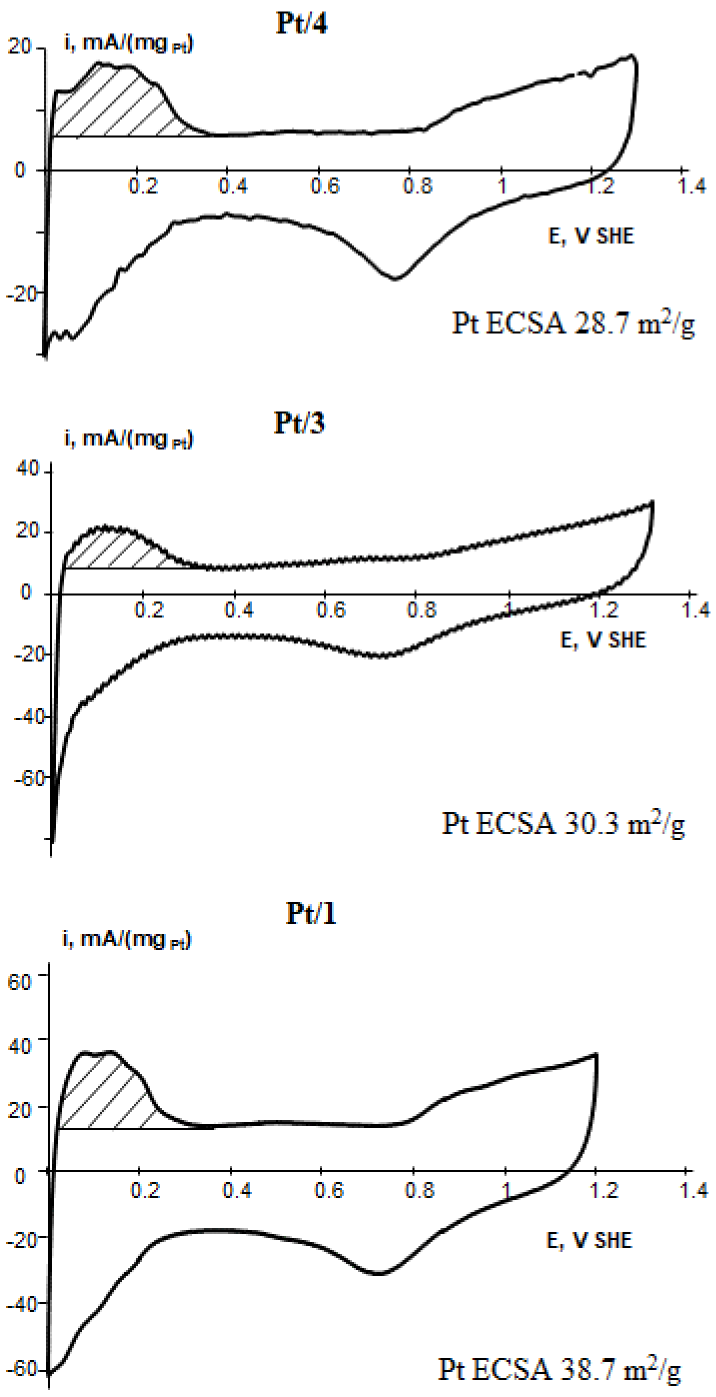

Figure S2. Cyclic voltammetry for platinated samples.

Polarization curve

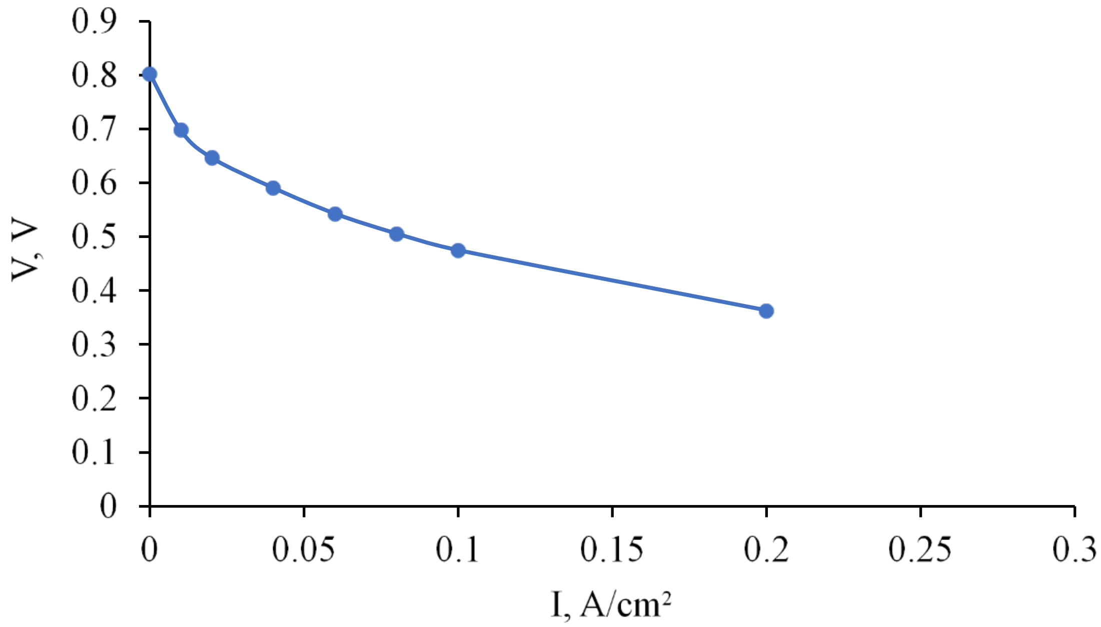

Figure S3. Polarization curve for sample **Pt/3**.

XPS studies

Table S1. Binding energies (BE), Gaussian widths (GW) and relative intensities (I_{rel}) of some groups for the C 1s spectra of the samples investigated.

Sample	Groups	C-C/C-H	C-M	sp^2	sp^3, C-N	C-OH, C-O-C	O-C-O	C(O)N	**C**(O)O
	Peak	C1	C2	C3	C4	C5	C6	C7	C8
**3**	BE, eV	281.96	283.10	284.44	285.55	286.66		288.05	
	GW, eV	0.9	0.9	1.0	0.89	1.0		1.2	
	I_{rel}	0.01	0.03	0.86	0.06	0.03		0.02	
**Pt/3**	BE, eV	282.0	283.2	284.44 (284.4)	285.45	286.6		288.0	
	GW, eV	0.9	0.9	0.85	0.89	1.0		1.2	
	I_{rel}	0.1	0.03	0.75 (0.08)	0.08	0.03		0.02	
**4**	BE, eV	282.35	283.16	284.44	284.89	285.72	286.94		288.42
	GW, eV	1.08	0.81	1.0	0.89	1.06	1.25		1.25
	I_{rel}	0.01	0.02	0.71	0.1	0.09	0.04		0.02
**Pt/4**	BE, eV		283.35	284.44	285.27		286.94		288.42
	GW, eV		0.9	1.0	1.1		1.3		1.3
	I_{rel}		0.04	0.59	0.24		0.08		0.04
	BE, eV				284.8	286.3	287.8		289.4
**1**	GW, eV			1.0	0.98	0.98	0.98	1.2	1.2
	I_{rel}			0.57	0.23	0.1	0.04	0.04	0.02
	BE, eV			284.44	284.72	285.78	286.79	287.81	289.27
**Pt/1**	GW, eV			1.0	1.01	0.98	0.98	1.2	1.2
	I_{rel}			0.57	0.24	0.11	0.04	0.04	0.01
	BE, eV			284.44	285.07	285.78	286.79	288.02	289.39
**Pt/1’**	GW, eV			1.0	0.98	0.98	0.98	1.2	1.2
	I_{rel}			0.48	0.22	0.16	0.08	0.04	0.02
	BE, eV				284.8	286.2		287.6	289.4
**2**	GW, eV				1.63	1.43		1.5	1.36
	I_{rel}				0.68	0.21		0.08	0.03

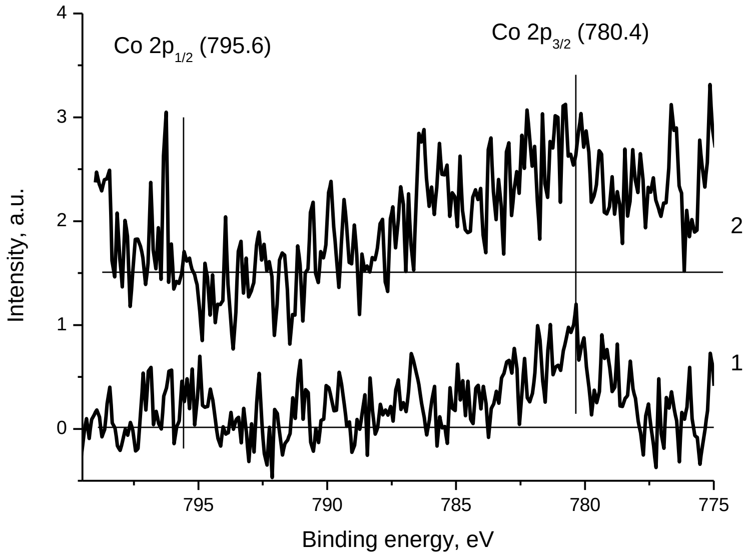

Fig. S4. Energy dependence of photoelectron emission in the energy region corresponding to Co 2p spectrum for the samples of **3** (1) and **Pt/3** (2).

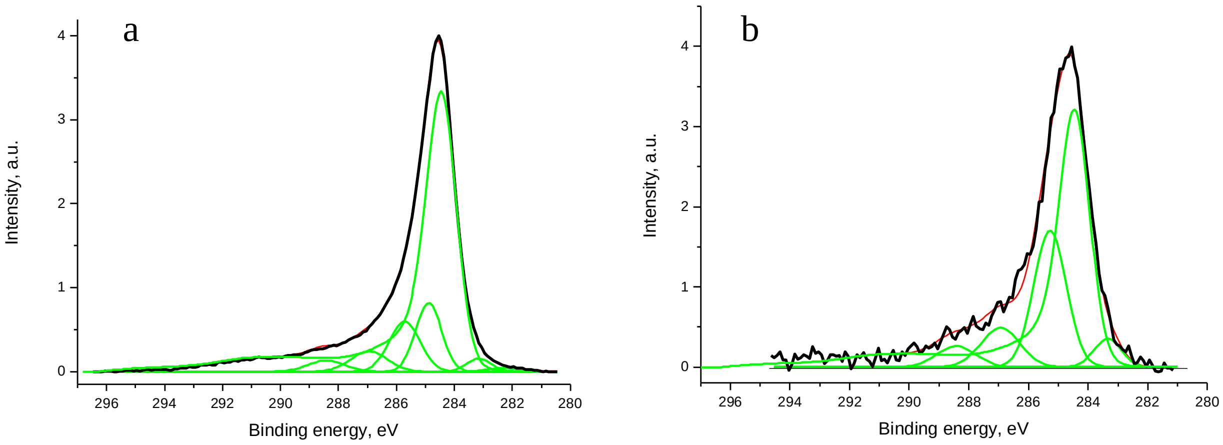

Fig. S5. The C 1s photoelectron spectra of samples **4**(a) and **Pt/4** (b).

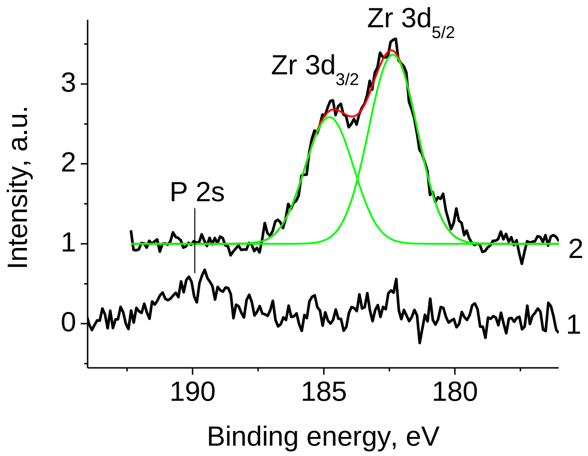

Fig. S6. The Zr 3d and P 2s photoelectron spectra of samples **Pt/4** (1) and **4** (2).

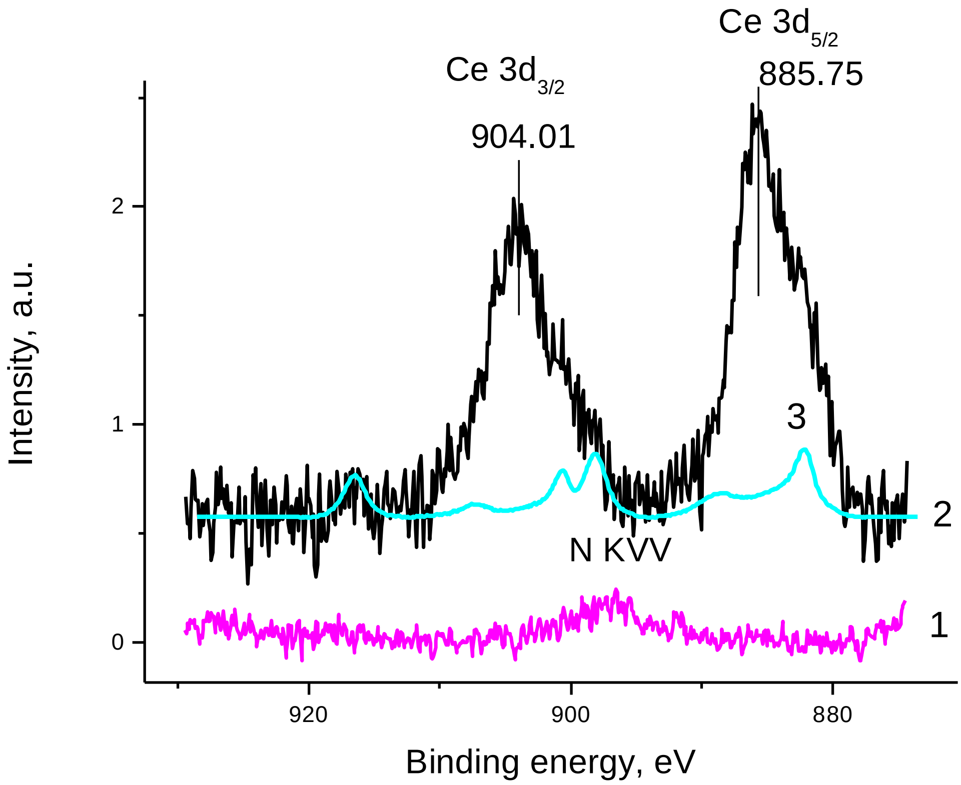

Fig. S7. N KVV Auger spectrum of sample **Pt/4** (1), photoelectron Ce 3d spectrum of sample **4** (2) and CeO_2 (3).

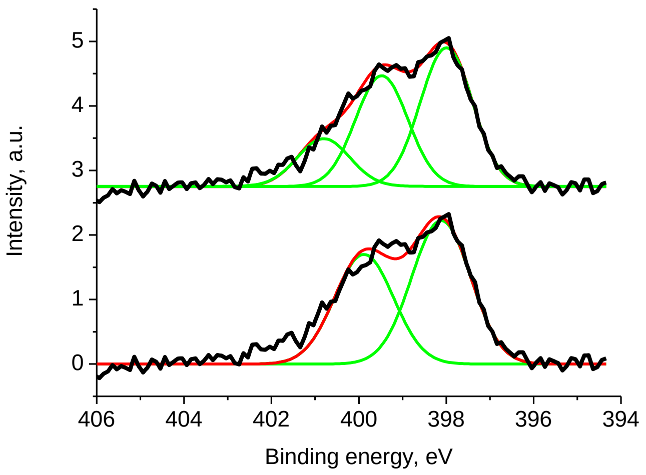

Fig. S8. The N 1s spectrum of sample **Pt/4** fitted with two and three peaks.

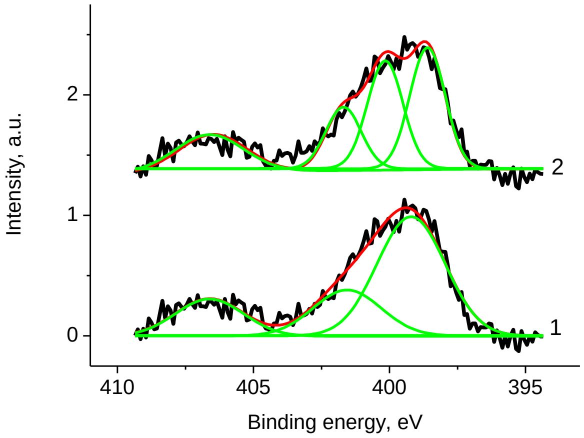

Fig. S9. The N 1s spectrum of sample **4** fitted with three (1) and four (2) peaks.

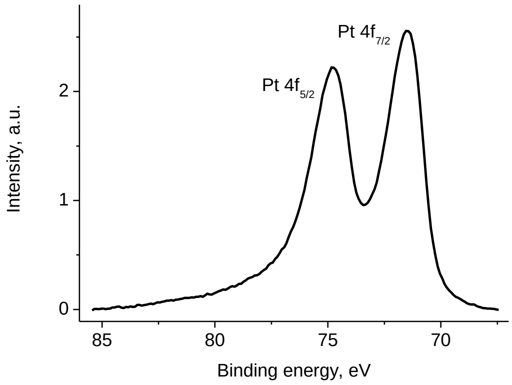

Fig. S10. Photoelectron Pt 4f spectrum of sample **Pt/4**.

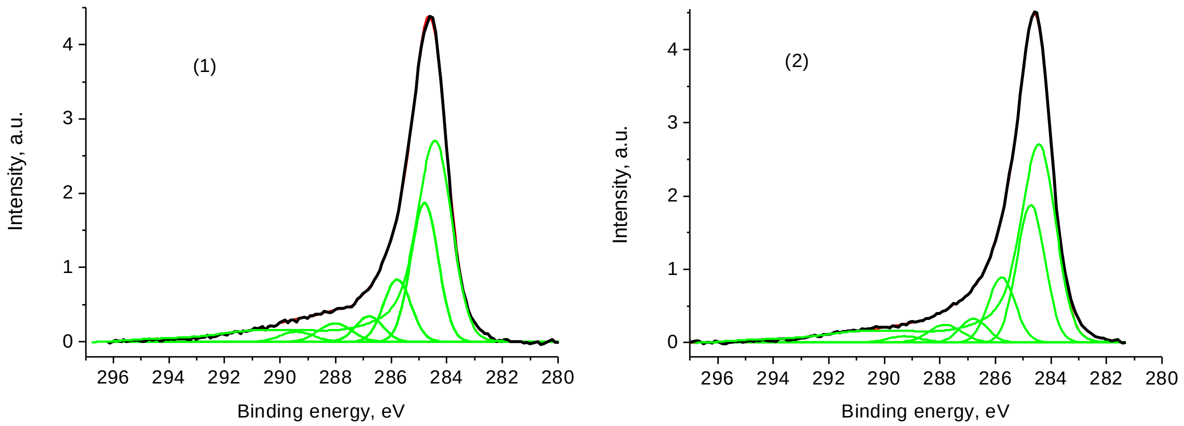

Fig. S11. The C 1s photoelectron spectra of samples **1** (1) and **Pt/1** (2).

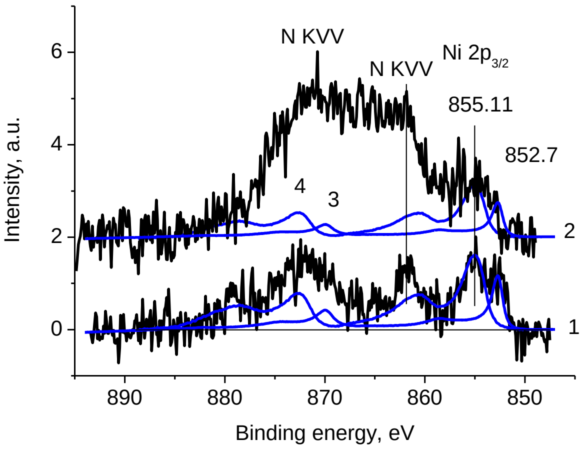

Fig. S12. The Ni 2p photoelectron spectra samples **1** (1), **Pt/1** (2) Ni foil (3) and sample $Ni(OH)_2$ (4).

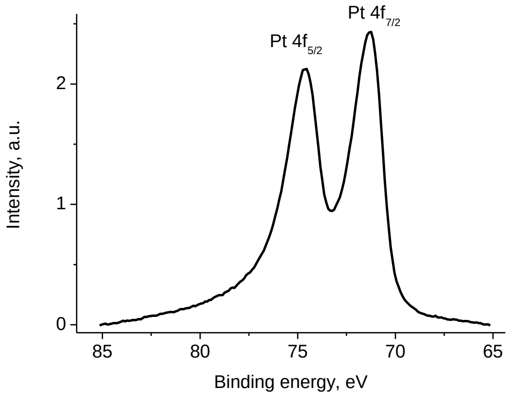

Fig. S13. The Pt 4f photoelectron spectrum of sample **Pt/1**.

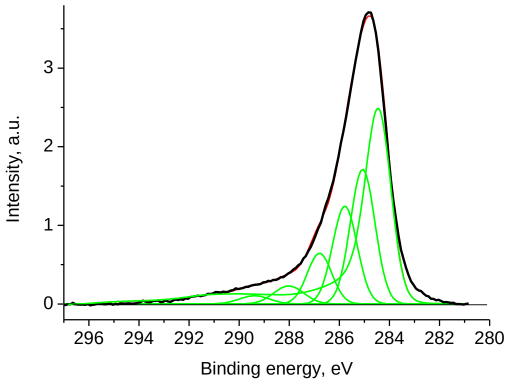

Fig. S14. The C 1s photoelectron spectrum of sample **1/Pt’**.

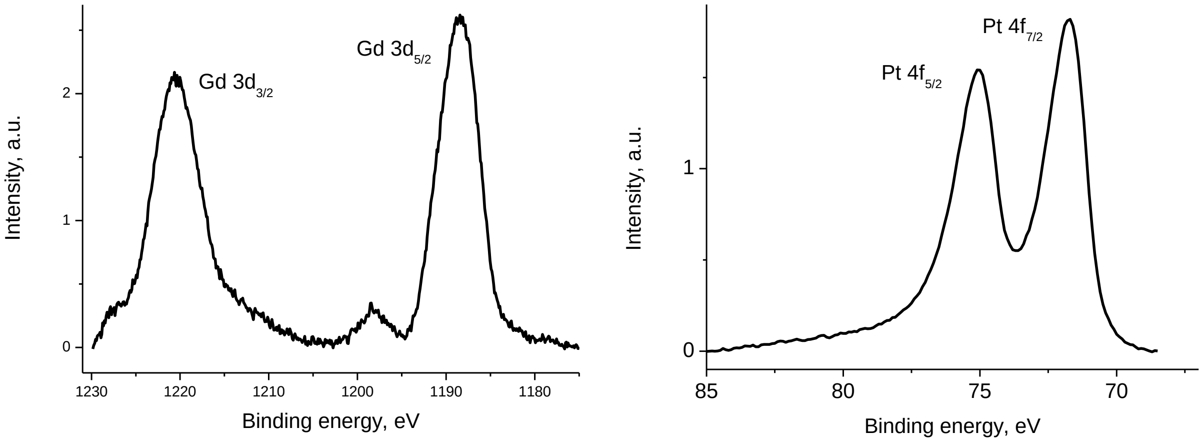

Fig. S15. The photoelectron Gd 3d and Pt 4f spectra of sample **Pt/1’**.

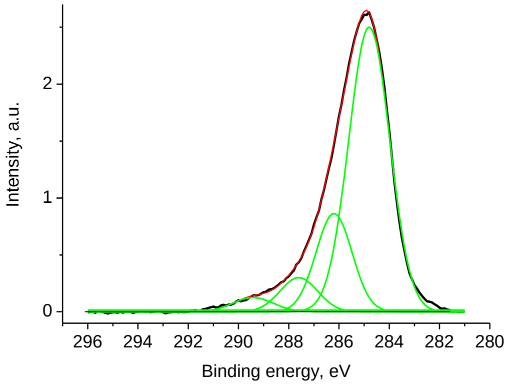

Fig. S16. The C 1s photoelectron spectrum of sample **2**.
